# PARalyzer: definition of RNA binding sites from PAR-CLIP short-read sequence data

**DOI:** 10.1186/gb-2011-12-8-r79

**Published:** 2011-08-18

**Authors:** David L Corcoran, Stoyan Georgiev, Neelanjan Mukherjee, Eva Gottwein, Rebecca L Skalsky, Jack D Keene, Uwe Ohler

**Affiliations:** 1 Institute for Genome Sciences and Policy, Duke University, 101 Science Drive, CIEMAS 2171, Box 3382, Durham, NC 27708, USA; 2 Program for Computational Biology and Bioinformatics, Duke University, 102 North Building, Durham, NC 27708, USA; 3 Department of Microbiology-Immunology, Feinberg School of Medicine, Northwestern University, 310 E. Chicago Ave, Chicago, IL 60611, USA; 4 Department of Microbiology-Immunology, Feinberg School of Medicine, Northwestern University, 320 E. Superior, Chicago IL 60611, USA; 5 Department of Molecular Genetics and Microbiology, Duke University Medical Center, 268 CARL Building, Box 3054 DUMC, Durham, NC 27710, USA; 6 Department of Biostatistics and Bioinformatics, Duke University, 2424 Erwin Road, Suite 1102 Hock Plaza, Box 2721, Durham, NC 27710, USA

## Abstract

Crosslinking and immunoprecipitation (CLIP) protocols have made it possible to identify transcriptome-wide RNA-protein interaction sites. In particular, PAR-CLIP utilizes a photoactivatable nucleoside for more efficient crosslinking. We present an approach, centered on the novel PARalyzer tool, for mapping high-confidence sites from PAR-CLIP deep-sequencing data. We show that PARalyzer delineates sites with a high signal-to-noise ratio. Motif finding identifies the sequence preferences of RNA-binding proteins, as well as seed-matches for highly expressed microRNAs when profiling Argonaute proteins. Our study describes tailored analytical methods and provides guidelines for future efforts to utilize high-throughput sequencing in RNA biology. PARalyzer is available at http://www.genome.duke.edu/labs/ohler/research/PARalyzer/.

## Background

RNA binding proteins (RBPs) play important roles in the life cycle of a transcript, from its nascence by RNA polymerase until its decay by RNases. All steps of RNA processing and function, including splicing, nuclear export, localization, stability, and small RNA-mediated regulation, are controlled by different RBPs and ribonucleoproteins [1]. The identification of which RBPs or ribonucleoproteins interact with which transcripts, how they interact, and where the interaction occurs, has been the focus of many studies. Recent advancements in high-throughput genomic technologies have resulted in profiles of transcriptome-wide RNA-protein interactions *in vivo*. Two of the most established methods for the investigation of these interactions are RIP-Chip [2] or RIP-seq [3,4] and crosslinking and immunoprecipitation (CLIP) [5]. RIP-Chip was the first method to use immunoprecipitation to identify RNA targets bound by specific RBPs at genome-wide scale [6]. Associated mRNAs are isolated, and then quantified using mRNA arrays or, more recently, subjected to high-throughput sequencing. This allows for the identification of all transcripts targeted by a particular RBP, but not for direct identification of where, or how many, RNA-protein interactions occur within a transcript. The second method, CLIP, typically uses short wave UV 254 nm crosslinking followed by immunoprecipitation and partial RNase digestion of the bound transcript. Conversion of the residual RNA segments into cDNA libraries and characterization by high-throughput sequencing yields small size windows in which the RNA-protein crosslinking occurred.

PAR-CLIP (photoactivatable-ribonucleoside-enhanced crosslinking and immunoprecipitation) is a powerful modification of the CLIP technology for the isolation of protein-bound RNA segments [7]. Cells are first cultured with a photoreactive ribonucleoside analogue, typically 4-thiouridine (4SU), to boost RNA-protein crosslinking. This is followed by high-throughput sequencing of cDNAs generated from the crosslinked immunopurified RNA fragments. During cDNA generation, preferential base pairing of the 4SU crosslink product to a guanine instead of an adenine results in a thymine (T) to cytosine (C) transition in the PCR-amplified sequence, serving as a diagnostic mutation at the site of contact. The pattern of T = > C conversions, coupled with read density, can thus provide a strong signal to generate a high-resolution map of confident RNA-protein interaction sites.

Here we present a new strategy specific for analysis of PAR-CLIP data to generate a transcriptome-wide high-resolution map of RNA-protein interaction sites. Our new method, dubbed PARalyzer, is designed to exploit the T = > C conversions introduced by the PAR-CLIP technology to generate high-resolution interaction sites that contain RBP binding sites with a strong signal-to-noise ratio. Combining PARalyzer interaction site identification with the motif-finding algorithm cERMIT [8], which is tailored to the analysis of high-throughput quantitative genomic data, reliably identifies the enriched common sequence patterns. Together, these two steps can be used to elucidate the transcriptome-wide set of RBP-mRNA interaction sites as well as the preferential binding motifs of the factors. We demonstrate the benefits of this approach on four published datasets, and provide guidelines and strategies for the analysis of future PAR-CLIP datasets. Both of these stand-alone command-line tools are available online [9].

## Results

### PAR-CLIP datasets

We focused our analysis on human PAR-CLIP datasets described in Hafner *et al*. [7], which profile the targets of four distinct mRNA-interacting factors. Three of the datasets were generated from immunoprecipitation data of the sequence-specific RBPs Quaking (QKI), Pumilio2 (PUM2), and Insulin-like growth factor 2 binding protein 1 (IGF2BP1). While QKI is a well-studied splicing factor in the nucleus [10], Pumilio RBPs are involved in mRNA stability and translation in the cytoplasm [11]. The functions of Pumilio are widely studied in a variety of species, and its global RNA targeting properties has been examined across a large phylogeny [12-17]. IGF2BP1 belongs to a family of proteins that are able to regulate translation by their direct binding to target mRNAs [18].

The fourth dataset consists of pooled libraries assaying members of the Argonaute (AGO) family of RBPs, central components of the RNA-induced silencing complex (RISC), which directs microRNAs (miRNAs) to their target transcripts, thereby negatively impacting gene expression [19]. Different from the other RBPs, Argonaute members do not have a specific mRNA recognition site; rather, their targets are specified by the interaction of the miRNA in RISC with partially complementary sequences in the target mRNAs [19]. The seed region of the miRNA is regarded as the important sequence determinant in target mRNA interactions [20]. AGO crosslinking is currently a popular method to directly identify miRNA targets, but the libraries contain a mixture of all targets of those miRNAs expressed in a particular cellular context.

Evaluating datasets for proteins with known sequence preferences allowed us to compare the interaction sites identified by PARalyzer with baseline methods, in terms of the presence of putative binding motifs normalized to the total size of the identified interaction sites. Initial analysis of PAR-CLIP data revealed that interaction sites of different proteins exhibit particular patterns of T = > C conversions, likely reflecting the accessibility of nucleotides in the RNA bound by the protein. Therefore, conversions do not have to include all thymines of a sequence motif equally, and may not even fall directly on top of conserved motifs at the interaction sites. Most notably, miRNA seed matches were observed to be largely devoid of T = > C conversions, and conversions were predominantly located directly upstream of the seed match.

### Methodology overview

T = > C conversion events that occur at the site of RNA-protein crosslinking can be used to identify the actual RBP interactions at high resolution, and subsequently, which sequence motifs are found at or close to these interaction sites. We have developed a toolkit that employs a non-parametric kernel-density estimate classifier, PARalyzer (PAR-CLIP data analyzer), to identify the RNA-protein interaction sites from a combination of T = > C conversions and read density. In a second step, PARalyzer interaction sites can be provided to *de novo *motif finders to elucidate sequence preferences; we adapted our recently published cERMIT algorithm for this task, and for the analysis of AGO libraries as an important special case.

### PARalyzer

Reads are first aligned to the genome, and those overlapping by at least a single nucleotide are grouped together. To exploit available read data in an effective way, we utilize relatively lenient alignment parameters. We allow reads to be as short as 13 nucleotides after adapter stripping, and a read may contain up to 2 mismatches restricted to T = > C conversions (in comparison, the analysis by Hafner *et al*. [7] used a read length of at least 20 nucleotides, and allowed for one T = > C mismatch). Within each read-group, PARalyzer generates two smoothened kernel density estimates, one for T = > C transitions and one for non-transition events. Nucleotides within the read groups that maintain a minimum read depth, and where the likelihood of T = > C conversion is higher than non-conversion, are considered interaction sites.

**Figure 1:**
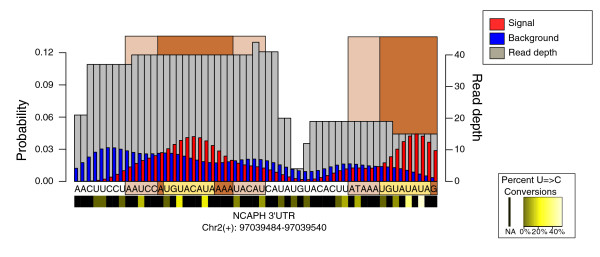
**Example of PARalyzer interaction site identification**. The entire genomic region corresponds to a single read-group from the Pumilio2 library. The orange region represents the nucleotides where the signal kernel density estimate is above background. The light pink locations are the full interaction sites extended by up to 5 nucleotides. A light gold box highlights the sequences that match the known Pumilio2 binding motif.

**Figure 2:**
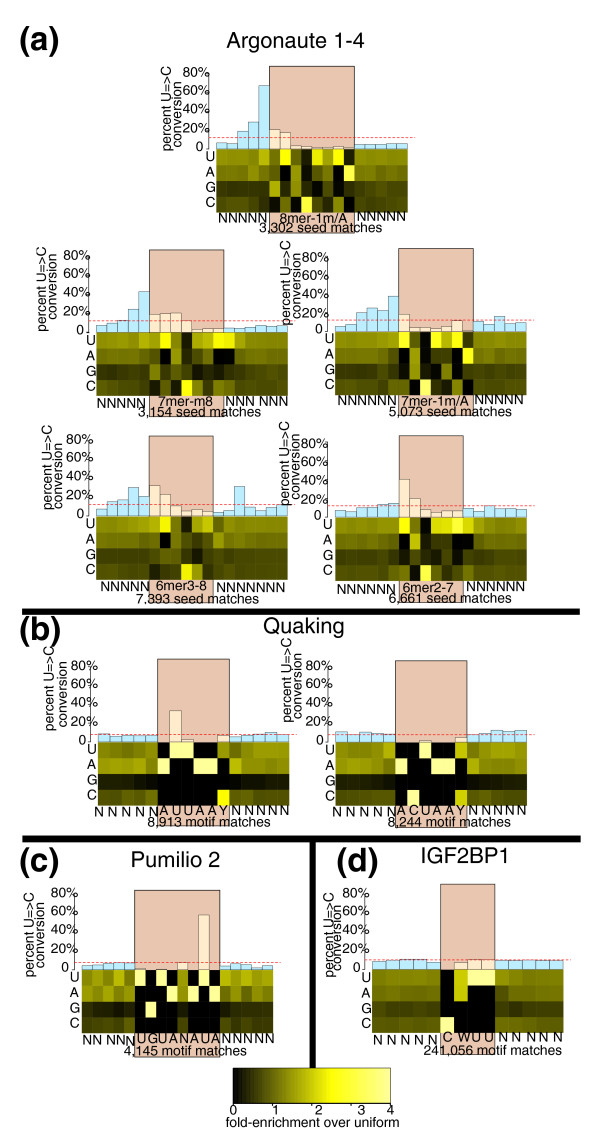
**Nucleotide composition and RNA crosslinking likelihood centered on AGO1-4, QKI, PUM2, and IGF2BP1 interaction sites**. The interaction site analysis is from all of the datasets: Quaking (QKI), Pumilio2 (PUM2), Insulin-like growth factor 2 binding protein 1 (IGF2BP1), and Argonaute 1 to 4 (AGO1 to -4). Heatmap: nucleotide composition, relative to a uniform background, of each individual binding site found in the respective genic regions. Barplot: likelihood of a T = > C conversion given that there is a 'T' at the given position. Unlike the heatmap, the barplot is not normalized by the number of reads mapping to an individual binding site. The red dotted line indicates the background conversion probability for all 'T's within the respective genic regions for each respective dataset. **(a) **Non-redundant seed-matches in 3' UTRs for the top 20 expressed miRNAs in the Argonaute dataset. 8 mer-m1 is a seed-match between the mRNA and nucleotides 1 to 8 of the miRNA seed sequence, 8 mer-A1 matches nucleotides 2 to 8 of the seed sequence paired with an A at position 1. 7 mer-1 m and 7 mer-A1 are similarly defined for nucleotides 1 to 7; 7 mer-m8 is a match utilizing nucleotides 2 to 8 of the seed sequence. 6 mer2-7 is a match utilizing nucleotides 2 to 7 of the seed sequence, and 6 mer3-8 utilizes nucleotides 3 to 8 of the sequence. **(b) **Motif matches for the two Quaking motifs in 3' UTRs, 5' UTRs, coding regions and introns. **(c) **Motif matches for the Pumilio 2 dataset in 3' UTRs, 5' UTRs, coding regions and introns. **(d) **Motif matches for the IGF2BP1 dataset in 3' UTRs, 5' UTRs, coding regions and introns.

Initial interaction sites are extended either to encompass the full underlying reads that contain a conversion event or by a generic window size (an example for the PUM2 dataset can be seen in Figure 1). The choice between these methods is dependent on the crosslinking properties of the analyzed RBP. For example, extending the region by five nucleotides on each side efficiently captures PUM2 binding sites, where crosslinking occurs directly at the motif. In contrast, when assaying the Argonaute protein family in which the miRNA-mRNA interaction site is protected from both digestion and T = > C conversion events, extending the region based on the underlying reads will include the location of conversion as well as the bound site, that is, the miRNA seed matches (Figure 2).

#### Motif finding

When sequence preferences are known, PARalyzer interaction sites can be examined for matches to the binding motif of the assayed factor. However, the majority of RBPs do not have known binding motifs. Furthermore, only a subset of miRNAs are expressed in any given cell type and available to be incorporated into the RISC. For the purposes of motif finding, current PAR-CLIP datasets fall into two distinct scenarios: (1) 'single binding motif analysis' in the case of sequence-specific RBPs (for example, QKI, PUM2, IFG2BP1); and (2) 'multiple motif analysis' in the special case of miRNA-mediated AGO-RNA crosslinking.

For the single binding motif analysis we apply the conserved Evidence Ranked Motif Identification Tool (cERMIT) [8], which was designed for *de novo *motif discovery based on high-throughput binding data (for example, ChIP-seq) and has been shown to exhibit highly competitive performance in the context of transcription factor binding site discovery [8]. There are two essential components of the motif discovery algorithm implemented by cERMIT: an enrichment function to score evidence of binding for a given sequence motif represented as a k-mer over the alphabet of IUPAC symbols 'A, C, G, U, W, K, R, Y, S, M, N'; and a search strategy that explores the motif space for high-scoring motifs. cERMIT differs from most other motif identification tools by making use of the complete quantitative evidence for a genome-wide set of regulatory regions. Rather than identifying a motif overrepresented in a pre-specified number of top candidate sequences, cERMIT ranks all putative target regions based on their binding evidence and identifies sequence motifs of flexible length that are highly enriched in targets with high binding evidence.

cERMIT is based on the assumption that evidence was available for an input set of potential regulatory target regions, independent of a specific analyzed factor (for example, all upstream regions for small genomes such as *Saccharomyces cerevisiae*, or regions of open chromatin in higher eukaryotes). Here, the regions to be evaluated are the PARalyzer interaction sites that are assigned evidence of RBP crosslinking. The binding evidence for PARalyzer-generated interaction sites is reflected in the number of observed (log2-transformed) T = > C conversions. In the data analyzed here, the number of observed T = > C conversions correlated well with the total number of reads (Additional file 1), which suggested that the motif finding strategy can also be applied to CLIP-seq datasets [5] by using the (log2 transformed) number of reads as binding evidence for each interaction site.

In the context of multiple motif analysis of AGO data sets we take advantage of the well-established mechanism of miRNA-based gene regulation [20,21], which is largely based on the 5' complementarity of miRNAs to target mRNA transcripts. Instead of performing a *de novo *motif search, the microRNA Enrichment Analysis Tool (mEAT) thus limits the search to a pre-specified seed list of known miRNAs, for example, as defined in miRBase [22]. In particular, we represent each miRNA by a short list of canonical end seed types: 8 mer-A1, 8 mer-m1, 7 mer-A1, 7 mer-m1, 7 mer-m8, 6 mer2-7, and 6 mer3-8. By rephrasing the original motif scoring within a classical linear regression framework, we can additionally allow for flexible and easily extensible accounting of biases unrelated to miRNA mediated AGO-mRNA interaction, such as sequence composition or interaction site size.

### Delineation of individual binding sites for sequence-specific RNA-binding proteins

**Figure 3:**
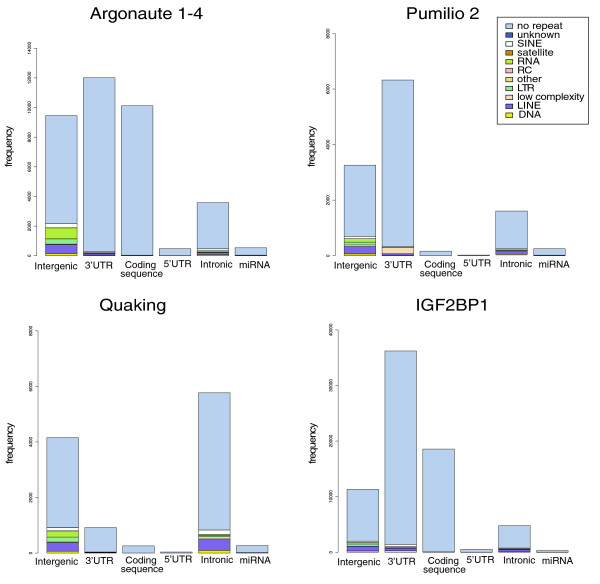
**Genomic location of PARalyzer generated interaction sites for four RNA-binding proteins**. Locations of interaction sites that contained at least two T = > C conversions were compared to transcript sequences as annotated in ENSEMBL (release 57) [42]. The different repeat region classes were identified by RepeatMasker [44]. The following repeat types were collected for this analysis: low complexity repeat family (low complexity), long interspersed nuclear elements (LINE), short interspersed nuclear elements (SINE), DNA transposons (DNA), RNA repeat families (RNA), satellite repeat family (Satellite), rolling circle (RC), unknown repeat family (Unknown), long terminal repeats (LTR) and other repeats (Other).

After applying PARalyzer to the four PAR-CLIP datasets described above, we observed that most of the interaction sites fell in the genomic regions expected for each of the different factors (Figure 3). The majority of Argonaute interaction sites were found in 3' UTRs, the region known to contain functional targets of the miRNA-associated RISC [19]. Similarly, the largest number of interaction sites was found in 3' UTRs for both Pumilio2 and IGF2BP1. Pumilio2 is a known regulator of mRNA translation and stability, which is facilitated by its binding to target gene 3' UTRs (reviewed in [17]). IFG2BP1, though less studied than Pumilio2, has also been shown to regulate translation and stability by binding either the 3' UTR or 5' UTR of its target genes [18,23]. In contrast, the majority of interaction sites found for Quaking, a known splicing regulator, were found in intronic regions [10].

A previously described baseline approach for the identification of interaction sites used groups of overlapping reads that contained at least a single T = > C conversion event [7], with more confident interaction sites being defined as those with higher numbers of T = > C conversion events. Reads had to be at least 20 nucleotides long, and contain at most one mismatch corresponding to a T = > C conversion. Our more lenient mapping parameters generally led to a larger number of initial read groups for each of the RBPs, but the number of interaction sites remained approximately the same for each dataset at a required read depth of 5. For the PUM2 dataset, we applied PARalyzer with the parameter option that extended the interaction sites by five nucleotides on each side of the positive signal. A comparison of the PUM2 results showed a 33% increase in the signal-to-noise ratio for the PARalyzer method (Table 1). Had we used the baseline parameter option of extending the interaction sites based on the underlying reads, we would have still seen a 20% increase in the signal-to-noise ratio. PARalyzer identified approximately the same number of motif instances, but interaction sites contain 29% fewer nucleotides.

**Table 1:** Summary of motif matches in the different PAR-CLIP datasets

	Number of motif matches	Total nucleotides	Signal-to-noise	Number of interaction sites with motif/Total number of interaction sites
Argonaute (top 20 expressed miRNAs)				
PARalyzer	3,933	207,334	2.68	3,041/11,353
Hafner *et al*. (CCRs)	4,106	301,227	1.92	3,090/6,796
Background (3' UTRs)	131,741	18,602,068	-	-
PUM2				
PARalyzer	1,262	127,168	60.28	1,344/6,990
Hafner *et al*.	1,371	200,228	41.59	1,290/5,668
Background	113,478	689,309,457	-	-
QKI				
PARalyzer	3,001	155,237	19.19	2,771/5,361
Hafner *et al*.	2,593	127,201	20.24	2,079/3,903
Background	694,229	689,309,457	-	-
IGF2BP1				
PARalyzer	31,507	1,718,152	1.35	24,758/55,831
Hafner *et al*.	51,429	3,739,750	1.01	32,303/59,784
Background	9,343,410	689,309,457	-	-

The Argonaute results are specific to only the 3' UTR region and contain only non-redundant seed matches. Summary of the motif matches for Pumilio2 (PUM2), Quaking (QKI), and Insulin-like growth factor 2 binding protein 1 (IGF2BP1) were generated from the analysis of the full transcript of all genes, including 5' UTRs, 3' UTRs, introns and coding regions. The Hafner *et al*. [7] crosslink-centered regions (CCRs) are those provided in their manuscript.

The current biases of the PAR-CLIP protocol (notably, the identity of the single photoactivatable nucleoside, as well as the endonuclease used for digestion), and the particular biochemistry of protein-RNA interactions place some constraints on the PARalyzer method. In available datasets, a good example is the QKI motif, where the preferred crosslinking occurs at the second nucleotide from the 5' end of the motif; when that nucleotide is a 'U', crosslinking occurs at a very high frequency; when it is a 'C', however, we cannot observe this event (Figure 2b). Use of a different photoactivatable nucleoside would likely result in the capture of this particular variation of the binding motif. Another good example is the identified IGF2BP1 motif 'CWUU', for which there is no dominant conversion event within or at a close, consistent distance to the binding motif (Figure 2d). In these particular cases, the uridines that are found within the preferred binding motif are protected from crosslinking, or show no particular likelihood of crosslinking over the background. When situations like this arise, interaction sites cannot be tightened beyond the extend-by-read option; the best choice is to identify regions of crosslinking and then extend the interaction site based upon the underlying reads that showed at least one conversion. In the case of Quaking, our mapping strategy in combination with PARalyzer results in the identification of 16% more sites at a cost of 5% signal-to-noise. In contrast, we identify only about half the number of IGF2BP1 motif instances that are found in the Hafner *et al*. [7] study, but at a signal above the expected background (Table 1).

While we limited our signal-to-noise analysis to interaction sites that were located on protein coding genes, it did not go unnoticed that there were many sites that fell within intergenic regions in each of the datasets (Figure 3). Analysis of intergenic interaction sites that met the same stringency cutoffs used above revealed that the number of motif matches per nucleotide is only slightly lower than for those sites that fall within known transcripts for both PUM2 and IGF2BP1, while not being as high for QKI or AGO (Additional file 2). This suggests that the PAR-CLIP libraries contain reliable RBP-mRNA interactions in currently unanotated, possibly non-coding transcripts.

Even though we employed a more lenient mapping strategy than the initial study, we still only mapped approximately 28% of the reads in each of the libraries to the genome. By relaxing mapping parameters further, and allowing up to three mismatches not necessarily limited to T = > C conversions, we find that a large number of the additional interaction sites generated are located in repeat regions of the genome. This includes short and long nuclear elements as well as other non-coding RNA-based families, suggesting nonspecific pull-down of highly abundant non-coding RNAs. A smaller fraction of these interaction sites contain preferred sequence motifs, and requiring of multiple T = > C conversion locations results in the elimination of many of these regions from subsequent analysis (Additional file 3).

**Figure 4:**
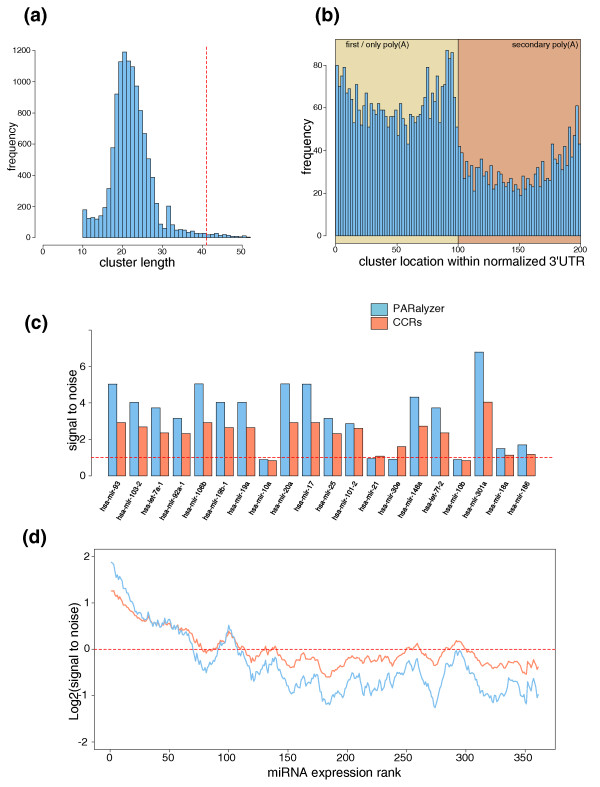
**Properties of Argonaute interaction site generation and their comparison to crosslink-centered regions**. **(a) **Distribution of interaction site sizes for the Argonaute dataset for sites that fall within 3' UTRs and contain two or more T = > C conversion locations. The vertical red line represents the 41-nucleotide size of the Hafner *et al*. [7] crosslink-centered regions (CCRs). **(b) **Distribution of interaction site locations across a normalized 3' UTR for all clusters that have two or more T = > C conversion locations. **(c) **The signal-to-noise for the top 20 expressed miRNAs in the Argonaute dataset for both PARalyzer generated interaction sites and the Hafner *et al*. [7] CCRs located in 3' UTRs. **(d) **Average log2 signal-to-noise ratio of window size 21 across all 361 miRNAs reported expressed in Hafner *et al*. in the order of their expression rank.

Overall, the PARalyzer method resulted in significant improvements. First, the size of the interaction site tends to be much smaller and therefore identifies sites at higher resolution (Figure 4a). Second, this approach can identify multiple sites within the same group of overlapping reads. Finally, our interaction sites never extend to regions that have zero read depth, as can be the case when selecting fixed-size windows around sites with observed conversion events. The simple approach of grouping reads leads to a strong influence of protocol (size selection) and/or sequencing technology (reliable read length), both of which should ideally not influence the identification of sites. The lenient short-read mapping in combination with PARalyzer thus provides a more comprehensive and higher resolution map of protein-RNA interaction sites. The method is easily adjustable when additional knowledge is available for the particular conversion pattern of an RBP. In any case, requiring at least two T = > C conversions in a read group is a strong indicator of the presence of binding for any RBP, even when lacking conversion directly at the consensus motif, possibly indicative of general non-site-specific interactions for stabilization of the RNA-protein interaction. This observation demonstrates the advantage of PAR-CLIP over other crosslinking protocols: even if conversions are not directly at the motif, they help to provide signal over noise.

### Examination of miRNA interaction sites

Different from sequence-specific RBPs, the baseline approach for the identification of Argonaute interaction sites in the PAR-CLIP study performed by Hafner *et al*. [7] was to use crosslink-centered regions (CCRs). CCRs are 41-nucleotide windows re-centered on the initial read group location that has the highest percentage of T = > C conversion events. A recent follow-up study suggested that CCRs could be used for all RBPs [24]. The 3' UTR is the specific region on a transcript where miRNA interactions have been shown to have the most significant impact on gene regulation [21,25]. Using PARalyzer, the signal-to-noise ratio of miRNA binding sites across 3' UTRs of genes known to be expressed in HEK293 cells was increased in the top expressed miRNAs (Table 1; Figure 4c); this ratio fell below the background level for miRNAs with very low or no expression in these samples (Figure 4d). A similar signal-to-noise ratio for seed-matches to the highly expressed miRNAs was observed for interaction sites within coding regions (Additional file 4). In contrast, the CCRs reported by Hafner *et al*. [7] led to lower signal-to-noise for highly expressed miRNAs, and remained close to the background level for lowly expressed miRNAs, indicating that the presence of seed motifs for these miRNAs was simply due to random matches in larger CCRs. This demonstrates that our method indeed created a higher resolution map of miRNA binding sites. Furthermore, conserved and putatively functional miRNA seeds have been reported to be located near the beginning of the 3' UTR and near poly-adenylation sites [26-28], and this pattern was confirmed for PAR-CLIP-derived binding sites (Figure 4b).

To examine crosslinking and conversion levels in more detail, we identified miRNA seed-matches for each of the top 20 expressed miRNAs within reads restricted to 3' UTRs or coding regions. Stratifying the interaction sites by canonical seed-match type resulted in the identification of distinct patterns of T = > C conversions (Figure 2a). For 8-mer and 7-mer matches, the highest likelihood of conversion fell one nucleotide upstream of the seed-match. The likelihood of a conversion event occurring within the seed-match tended to be at or below the background conversion rate. This confirmed previous observations that the miRNA-mRNA base pairing prevents crosslinking between the protein and any 4SU on the mRNA within the seed region, and that conversions largely fall just outside the seed region where Argonaute proteins are in close proximity to the single-stranded target mRNA molecule. Contrary to 8- and 7-mer matches, conversion events were more likely to occur within 6-mer seed matches than the surrounding area. These trends were also observed in seed matches identified in reads that map to coding regions (Additional file 4). While 6-mer matches are more likely to occur by chance, and some might be non-functional even when located in PAR-CLIP interaction sites, these differences may reflect structural transitions that are induced by more extensive seed pairing [29], altering the protein conformation and RNA crosslinking efficiency.

Several studies have pointed out that the nucleotide composition surrounding a miRNA binding site plays a role in that site's effectiveness to regulate the target gene [26,30], and in agreement, we observed that the nucleotides immediately adjacent to any type of seed match in 3' UTRs were AU rich (Figure 2a). While the overall AU content was high in 3' UTRs, it was lower in sites present in coding regions (Additional file 5), and normalizing for AU content of the different genomic regions reduced the effect. Interestingly, binding sites for the other RBPs (QKI, PUM2 and IGF2BP1) also occurred within AU-rich regions, with an under-representation of guanines surrounding the interaction sites. The latter may be due to the fact that the RNase T1 enzyme, used in the preparation of the analyzed PAR-CLIP libraries, preferentially cleaves next to Gs. Cleavage of Gs immediately surrounding the binding sites could result in short RNA fragments, too short in fact to be included in the library because of a read size selection step that specifically collects reads approximately 30 nucleotides in size. Given that the RBPs studied here protect a region of 6 to 12 nucleotides, fragments with Gs immediately next to the site are likely to be too short to pass the size selection step. Alternatively, it is also possible that the high AU richness of these binding regions is necessary for RBP accessibility.

### Evidenced-ranked *de novo *motif identification

Hafner *et al*. [7] successfully applied standard motif discovery approaches (PhyloGibbs [31], MEME [32]) on the subset of the top 100 most highly confident read-groups to predict RNA binding preferences. Choosing an arbitrary cutoff is well justified in cases where the target-binding motif is of low degeneracy and/or long and hence contains high discriminative signal relative to the background sequence. When this is not the case, a larger set of example sequences with the motif occurrence, with possibly variable binding affinity, can facilitate the search process.

For the single binding motif analysis we therefore used a recently developed method, cERMIT [8], which was specifically designed for *de novo *motif discovery based on high-throughput binding data (for example, ChIP-seq) and shown to exhibit highly competitive performance in the context of transcription factor binding site and miRNA seed discovery [8]. Motif identification on the QKI and PUM2 datasets was successful in recovering their respective reported consensus binding motifs [7,10,33] (Additional files 6 and 7). The motif for IG2BP1, which had not previously been identified, was highly similar to the one reported by Hafner *et al*. [7] (Additional file 8). For this analysis, we used all PARalyzer interaction sites mapping to a genic region not flagged as a repeat.

For the multiple motif analysis on the combined AGO PAR-CLIP datasets, we took all human miRNAs available in miRBase v16 as input for mEAT, which adapts cERMIT to a restricted motif analysis over miRNA seed matches. Despite starting from all known human miRNAs, our analysis automatically ranked the top expressed miRNAs in the cell line on the top of the list of predicted enriched miRNA seed clusters (Table 2). Therefore, this enrichment analysis can be used to identify those miRNAs with the strongest impact on mRNA targeting, even in the absence of miRNA expression information. While the initial PAR-CLIP study reported that seed matches could explain about 50% of CCRs, this was based on 6-mer matches to the top 100 expressed individual miRNAs. As our analysis above showed, only the matches of the top approximately 60 or so miRNAs provide a signal above background. The *de novo *motif analysis here confirms this: the top 5 expressed miRNAs alone can explain approximately 18% of all targets, but collectively, all 25 significantly enriched seed match families covered only approximately 30% of the interaction sites.

**Table 2:** Summary of the top *de novo *miRNA target predictions based on the Argonaute PAR-CLIP data

Cluster	miRbase ID	8-mer	Expression rank	miRNA score	*P*-value	Number of targets	Cumulative number of targets
1	hsa-mir-16-2	TGCTGCTA	22	17.93	3.0E-20	438	438 (3%)
	hsa-mir-15b	TGCTGCTA	53	17.93	3.5E-20	438	438 (3%)
	hsa-mir-15a	TGCTGCTA	64	17.93	3.5E-20	438	438 (3%)
	hsa-mir-195	TGCTGCTA	NA	17.93	3.5E-20	438	438 (3%)
	hsa-mir-16-1	TGCTGCTA	NA	17.93	3.5E-20	438	438 (3%)
	hsa-mir-103-2	ATGCTGCT	2	14.41	9.7E-13	620	620 (5%)
	hsa-mir-107	ATGCTGCT	39	14.41	9.7E-13	620	620 (5%)
	hsa-mir-103-1	ATGCTGCT	NA	14.41	9.7E-13	620	620 (5%)
	hsa-mir-424	TGCTGCTG	60	12.92	1.5E-08	632	632 (5%)
	hsa-mir-497	TGCTGCTG	133	12.92	1.5E-08	632	632 (5%)
	hsa-mir-646	AGCTGCTT	NA	10.5	1.1E-06	708	708 (6%)
	hsa-mir-503	CGCTGCTA	97	10.08	1.7E-07	714	714 (6%)

2	hsa-mir-106b	GCACTTTA	5	17.63	8.9E-17	455	1,164 (9%)
	hsa-mir-20a	GCACTTTA	9	17.63	8.9E-17	455	1,164 (9%)
	hsa-mir-106a	GCACTTTT	121	15.65	1.6E-15	565	1,272 (10%)
	hsa-mir-519c	TGCACTTT	NA	14.71	7.6E-21	689	1,395 (11%)
	hsa-mir-519c-3p	TGCACTTT	NA	14.71	7.6E-21	689	1,395 (11%)
	hsa-mir-519a-2	TGCACTTT	NA	14.71	7.6E-21	689	1,395 (11%)
	hsa-mir-519b-3p	TGCACTTT	NA	14.71	7.6E-21	689	1,395 (11%)
	hsa-mir-519a-1	TGCACTTT	NA	14.71	7.6E-21	689	1,395 (11%)
	hsa-mir-526bstar	GCACTTTC	NA	14.57	4.8E-22	746	1,450 (12%)
	hsa-mir-93	GCACTTTG	1	12.99	1.4E-13	790	1,490 (12%)
	hsa-mir-17	GCACTTTG	10	12.99	1.4E-13	790	1,490 (12%)
	hsa-mir-20b	GCACTTTG	NA	12.99	1.4E-13	790	1,490 (12%)
	hsa-mir-519d	GCACTTTG	NA	12.99	1.4E-13	790	1,490 (12%)
	hsa-mir-520d-3p	AGCACTTT	NA	12.15	4.2E-11	796	1,496 (12%)
	hsa-mir-520b	AGCACTTT	NA	12.15	4.2E-11	796	1,496 (12%)
	hsa-mir-520e	AGCACTTT	NA	12.15	4.2E-11	796	1,496 (12%)
	hsa-mir-372	AGCACTTT	NA	12.15	4.2E-11	796	1,496 (12%)
	hsa-mir-520c-3p	AGCACTTT	NA	12.15	4.2E-11	796	1,496 (12%)
	hsa-mir-520a-3p	AGCACTTT	NA	12.15	4.2E-11	796	1,496 (12%)
	hsa-mir-3609	TCACTTTG	NA	10.2	9.3E-09	798	1,498 (12%)

3	hsa-mir-92a-1	GTGCAATA	4	13.59	4.8E-10	223	1,709 (14%)
	hsa-mir-32	GTGCAATA	95	13.59	4.8E-10	223	1,709 (14%)
	hsa-mir-92b	GTGCAATA	101	13.59	4.8E-10	223	1,709 (14%)
	hsa-mir-92a-2	GTGCAATA	NA	13.59	4.8E-10	223	1,709 (14%)
	hsa-mir-25	GTGCAATG	11	11.38	2.2E-09	239	1,722 (14%)
	hsa-mir-363	GTGCAATT	130	11.33	1.6E-09	265	1,746 (14%)
	hsa-mir-367	GTGCAATT	NA	11.33	1.6E-09	265	1,746 (14%)

4	hsa-mir-454	TTGCACTA	108	12.04	2.3E-04	298	1,904 (16%)

5	hsa-mir-101-2	GTACTGTA	12	11.87	1.7E-11	202	2,098 (17%)
	hsa-mir-101-1	GTACTGTA	NA	11.87	1.7E-11	202	2,098 (17%)
	hsa-mir-144	ATACTGTA	NA	9.83	8.3E-06	260	2,151 (18%)

Clustering is based on highly similar miRNA seeds (third column). Predictions are ordered based on the enrichment scores assigned by the motif analysis performed using mEAT. For each cluster prediction we report the expression rank (fourth column), the mEAT enrichment score (fifth column), the *P*-value estimate based on permuting the binding evidence assignment (100 draws) combined with a parametric fit to a Gaussian distribution (sixth column), the number of targets that represents the total number of regions with a match to at least one of the canonical seeds of the cluster members (seventh column), and the cumulative number of targets that corresponds to the union of the predicted targets of the current cluster with all others preceding it (eighth column). miRNAs that were not reported as expressed in Hafner *et al*. [7] were assigned 'NA' values; some of these are recently identified miRNAs not known at the time of measuring expression levels.

## Discussion

As with many new short-read deep-sequencing protocols, the PAR-CLIP approach to elucidate RNA binding sites enables specific opportunities for in-depth analysis and interpretation of genomic data. In addition to mapping sequence-specific RBPs such as PUM2, QKI or IGF2BP1, an anticipated popular application of this protocol will be to study binding by members of the RISC, making it possible to identify the joint set of transcriptome-wide miRNA targets under specific conditions. To address the challenges posed by these two scenarios, we described the PARalyzer approach, which uses a kernel density estimate classification to generate a high-resolution map of RNA-protein interaction sites. In addition, we described an extension of our previous motif finding algorithm, cERMIT, to subsequently identify binding motifs for sequence-specific RBPs or over-represented miRNA seed matches.

Analysis of the Argonaute datasets showed that miRNA seed matches allowed for refining several previous findings on miRNA targeting. As reported, miRNA binding sites are located within AU-rich regions, but this was limited to sites in the 3' UTR; miRNA seed matches found in the coding regions of genes did not exhibit this nucleotide bias. While the overall number of interaction sites found in coding regions was smaller than in 3' UTRs, the signal-to-noise ratio of the identified coding interaction sites almost reached the levels at seed matches found in 3' UTRs. The evidence for binding alone obviously does not imply that these sites have similar functional consequences to those found within the 3' UTR. Confirming previous studies based on sequence or expression, but not direct binding, miRNAs were most likely to interact with their targets near the ends of the 3' UTRs, including alternative poly-adenylation sites.

A detailed study of sequence-specific RBPs (PUM2, QKI and IGF2BP1) revealed the strengths and current limitations of the PAR-CLIP protocol, and as a consequence, methods for the analysis of PAR-CLIP data. PUM2 data showed a high likelihood of T = > C conversion occurring directly at the RNA-protein interaction site and within the conserved binding motif. In such cases, our approach can identify the true transcriptome-wide interaction sites at (nearly) single nucleotide resolution. On the other hand, analysis of QKI data exhibited differences: while the 'AUUAAY' binding motif showed strong likelihood of T = > C conversion at a particular nucleotide in the recognition motif, the 'ACUAAY' motif had no specific site where a conversion event could be detected. In such cases, the lack of a particular location of conversion prevents single nucleotide resolution of the interaction site, and at first glance seems to erase the strengths of PAR-CLIP compared to standard CLIP data. However, requiring T = > C conversions to occur in the vicinity is still a good method to enrich for true binding sites: while no particular nucleotide near the binding motif exhibited conversion preferences, it suggested that non-specific, possibly stabilizing interactions of another component of the RBP with the RNA molecule gave PAR-CLIP an advantage over other *in vivo *RBP-RNA interaction detection protocols.

The different, and in many cases unknown, crosslinking properties for RBPs presents a challenge for all CLIP protocols, and requires small adjustments as to how to call and expand interaction sites to ensure the inclusion of the binding site. In instances of newly studied proteins, for which the motif or conversion pattern is not known-for example, the recently analyzed HuR protein [34]-it is thus best to use PARalyzer with the 'extend-by-read' option in combination with the output of motif finding to determine if significant top-scoring motifs tend to have specific locations of high conversion. If there is at least one location of high conversion, as is, for example, the case for PUM2, then a tighter extension can be used to reduce the size of the interaction map.

In addition to the RBP-specific sequence affinity preferences, the RBP-RNA interaction has been shown to be influenced by the secondary structure of the targeted RNA sequence and has been successfully exploited in previous work on RBP motif discovery [35-37]. Incorporating information on the RBP structural preferences into the motif analysis proposed in the current work could be implemented by means of a prior distribution on the binding evidence for individual sequence regions inferred by PARalyzer, biasing the motif discovery towards high-scoring sequence patterns that contain favorable sequence context for RBP binding. This could help filter out non-specific interactions with highly abundant mRNAs. In the context of AGO-mediated regulation, a prior based on the predicted miRNA-mRNA duplex stability could be used in a similar fashion

Due to the use of 4SU nucleoside analogue in the original PAR-CLIP protocol, the 'U' content of an actual binding site and its vicinity will obviously impact the identification of RBP binding sites. If a recognition site does not contain any uridines, precise delineation using this approach is compromised; on the other hand, many U residues may either cause problems with alignment due to the potential of many mismatches, and/or to spread out the signal over multiple positions. The current investigations of additional amenable photoactivatable nucleosides [38], complemented by the use of different digestion enzymes [24], are expected to reduce potential biases, and can easily be specified in PARalyzer. As such, our pipeline provides a standardized solution for the analysis of RBP binding sites via PAR-CLIP, for subsequent motif finding for sequence-specific RBPs, and for the elucidation of post-transcriptional regulatory mechanisms and networks.

## Materials and methods

### Processing, mapping, and grouping of short-read data

Short read libraries were downloaded from the Short Read Archive [39] (SRX020777, SRX020781-6). Reads from the deep sequencing libraries were first stripped of the 3' adapter sequence using the FASTX toolkit [40]. Reads that were less than 13 nucleotides in length or contained an ambiguous nucleotide were discarded. The remaining reads were aligned to the human genome (hg19) by the Bowtie algorithm [41], with up to two mismatches allowed. Mapped locations were only reported for the optimal mismatch-stratum for each read up to a maximum of ten different locations. All T = > C mismatches between a read and the genomic sequence were subtracted from the mismatch count at each mapped location. Only reads that mapped to a single genomic location with no mismatches after conversion subtraction were used for further analysis. The location that a read mapped to, relative to a known transcript, was determined based on the ENSEMBL database (release 57) [42]. If a read mapped to a location that could be placed in multiple categories, it was assigned based on the following order of preference: 3' UTR, coding sequence, 5' UTR, miRNA, intron, intergenic. Reads that overlapped by at least a single nucleotide were grouped together to form read groups. The location of a read group relative to known transcripts was determined in the same way as for individual reads. Original clusters and CCRs were obtained from Hafner *et al*. [7] and converted to hg19 coordinates using the liftover tool from the UCSC genome browser [43].

Repetitive sequence regions were identified by RepeatMasker [44] and the specific locations were downloaded from the UCSC genome browser [43]. The following repeat types were collected for this analysis: low complexity repeat family (low complexity), long interspersed nuclear elements (LINE), short interspersed nuclear elements (SINE), DNA transposons (DNA), RNA repeat families (RNA), satellite repeat family (Satellite), rolling circle (RC), unknown repeat family (Unknown), long terminal repeats (LTR) and other repeats (Other).

### Identification of motif matches

Motif matches for PUM2 were identified by a string search for 'UGUANAUA' in all read groups that were found in 3' UTRs, 5' UTRs, coding regions or introns. Local nucleotide composition around each site was determined by collecting ± 5 nucleotides from each binding site. The heatmap shown in Figure 2c includes each motif match, regardless of the number of reads that map to that particular location. The T = > C conversion graph associated with the heatmap is based on all reads that map at or around the motif match, and therefore is not normalized by the number of reads at any particular position. Motif matches and figures for the QKI and IGF2BP1 proteins (Figure 2b) were prepared in the same way, but with string searches for the Quaking motifs 'AUUAAY' and 'ACUAAY', or 'CAUU' and 'CUUU' for IGF2BP1.

Seed matches for miRNAs in the Argonaute dataset were collected for non-redundant matches to the top 20 expressed miRNAs. Expression rank of the miRNAs was provided in Hafner *et al*. [7] and was determined by deep sequencing libraries generated in parallel with the PAR-CLIP libraries. Seed-matches were identified in order from longest to shortest, and overlapping seed matches of different length were only included in the analysis of the longest possible seed match. The preference of seed match was searched in the following order: 8 mer-m1, 8 mer-A1, 7 mer-m1, 7 mer-A1, 7 mer-m8, 6 mer2-7, 6 mer3-8. 8 mer-m1 is a seed-match between the mRNA and nucleotides 1 to 8 of the miRNA seed sequence, 8 mer-A1 matches nucleotides 2 to 8 of the seed sequence paired with an A at position 1. 7 mer-1 m and 7 mer-A1 are similarly defined for nucleotides 1 to 7; 7 mer-m8 is a match utilizing nucleotides 2 to 8 of the seed sequence. 6 mer2-7 is a match utilizing nucleotides 2 to 7 of the seed sequence, and 6 mer3-8 utilizes nucleotides 3 to 8 of the sequence. The heatmaps and barplots for the different seed-match types (Figure 2a) were calculated in the same manner as those described for PUM2. Sites found to be targeted by multiple miRNAs in the top 20 expressed were only included once for the most highly expressed miRNA.

### PARalyzer

For each read group that contained at least five reads and two T = > C conversion locations, a kernel-density-based classifier was utilized to more precisely delineate the region of crosslinking ('signal') versus non-crosslinking ('background'). The minimum number of five reads was motivated by the need to reliably estimate the densities, and can be adjusted to higher numbers in more comprehensive sequence libraries. Class-specific densities were estimated using a Gaussian kernel density estimator with globally fixed precision parameter *λ *= 3. The signal-to-noise results are fairly robust to the setting of the bandwidth parameter (Additional file 9).

More formally, for a given read group of length L we define $x_{\text{T}\to \text{T}}^{\text{(i)}}$ and $x_{\text{T}\to \text{C}}^{\text{(i)}}\text{, i }\in \text{\{ 1,}...\text{,L\}}$ to be the number of observed conversion and non-conversion events, respectively, at an offset i relative to the start, and with a minimum read depth of 5 to be able to estimate conversion frequencies. The read depth is the number of individual reads that map to a region overlapping a particular nucleotide. Let n_T→T _and n_T→C _be the total number of conversion and non-conversion events in the group. For any position j ∈ {1,..., L} we define:

$$\begin{array}{c} {\text{f}}_{\text{T}\to \text{C}}\text{(j) = }\overset{\text{L}}{\underset{\text{i = 1}}{\sum }}\frac{{\text{x}}_{\text{T}\to \text{C}}^{\text{(i)}}}{{\text{n}}_{\text{T}\to \text{C}}}\times \frac{\text{1}}{\sqrt{\text{2}\lambda^{\text{2}}\pi }}{\text{e}}^{-\frac{{∥\text{i - j}∥}^{\text{2}}}{\text{2}\lambda^{\text{2}}}}\\ {\text{f}}_{\text{T}\to \text{T}}\text{(j) = }\overset{\text{L}}{\underset{\text{i = 1}}{\sum }}\frac{{\text{x}}_{\text{T}\to \text{T}}^{\text{(i)}}}{{\text{n}}_{\text{T}\to \text{T}}}\times \frac{\text{1}}{\sqrt{\text{2}\lambda^{\text{2}}\pi }}{\text{e}}^{-\frac{{∥\text{i}-\text{j}∥}^{\text{2}}}{\text{2}\lambda^{\text{2}}}} \end{array}$$

which, after normalization, produces a non-parametric estimate for the density of conversions and non-conversions, respectively:

$$\begin{array}{lll} {\text{k}}_{\text{T}\to \text{C}}\text{(j) = }\frac{{\text{f}}_{\text{T}\to \text{C}}\text{(j)}}{\sum_{\text{j}=\text{1}}^{\text{L}}{\text{f}}_{\text{T}\to \text{C}}\text{(j)}} & \; & \text{(1)}\\ {\text{k}}_{\text{T}\to \text{T}}\text{(j)}=\frac{{\text{f}}_{\text{T}\to \text{T}}\text{(j)}}{\sum_{\text{j}=\text{1}}^{\text{L}}{\text{f}}_{\text{T}\to \text{T}}\text{(j)}} & \; & \text{(2)}\\ & \; & \text{(3)} \end{array}$$

Nucleotide positions j for which k_T→C_(j) > k_T→T_(j) are considered to be interaction sites.

Interaction sites derived from the PUM2 read groups were extended up to five nucleotides in each direction as long as a minimum read depth of 5 was maintained; sites that overlapped upon this extension were combined into a single interaction site. Interaction sites for Argonaute, QKI and IGF2BP1 were extended in both directions, up to the most distal end of reads that overlapped the interaction site by at least one nucleotide and had at least a single T = > C conversion event; extension in either direction was halted where the read depth fell below the cutoff of five reads, and interaction sites that overlapped were joined to a single site. This is considered the 'extend-by-read' option. This extension strategy was suitable for the identification of miRNA binding sites because crosslinking events were observed to occur adjacent to the seed match and not directly at the interaction site. This approach also worked for QKI and IGF2BP1 factors because the conversions happened near the site, despite Us within their binding motifs being protected from crosslinking. We did not believe an interaction site should be called based on only a single read as done previously; however, PARalyzer maintains a similar signal-to-noise ratio when analyzing read-groups that contain at least five reads, but not necessarily at a depth of 5 at any one position (Additional file 10). Locations of interaction sites relative to known transcripts were determined in the same manner as for individual reads.

### Signal-to-noise estimation

Hafner *et al*. [7] utilized HGU133 Plus 2.0 microarrays to determine the expression value for all known genes in the HEK293 cell line for comparison with their PAR-CLIP experiments. All probes were assigned to genes according to ENSEMBL release 57 when possible. The average value of two biological replicates was used for each probe. When multiple probes were available for the same gene, the highest expression value was used for that particular gene. All genes with expression values above the 80th percentile of all background probes (those that were not associated with a gene) were considered expressed.

Signal-to-noise was calculated as the number of sites per nucleotide in a given set of read-groups, CCRs, or PARalyzer interaction sites, divided by the sites per nucleotide in the background set. For PUM2, IGF2BP1 and QKI, the complete gene sequences for all protein coding genes were used as background set (UTRs, coding sequence and introns as identified in ENSEMBL release 57 [42]). In instances where multiple isoforms are known for a specific gene, the sequence for the longest transcript was used. For the Argonaute miRNA analysis, we used the sequence of the 3' UTRs, or separately, the set of all coding regions. For instances where more than one 3' UTR was identified for a specific gene, the sequence for the longest 3' UTR was used; the same approach was applied for coding sequences. Signal-to-noise was calculated from only those genes that were identified as expressed by the microarray analysis.

### Alternative 3' UTRs

Coordinates of experimentally verified 3' UTRs were collected from PolyA_DB (version 2) [45] and additionally curated as previously described in Majoros and Ohler [27]. Genes found in both PolyA_DB and ENSEMBL release 57 were used in further analysis. Each 3' UTR was normalized to a length of either 100% or 200%, based on whether the gene had one or two or more annotated poly-adenylation sites. The midpoint of the interaction site was used as reference location within the normalized 3' UTR.

### Evidence-ranked motif identification in PAR-CLIP data

Let s_i_, i ∈ {1,..., n} be a set of sequence regions (for example, interaction sites or read groups as reported by PARalyzer) and y_i _be the corresponding binding evidence for each region (here, log2[#T = > C]). We define the candidate set of putative motifs to be m_j_, j ∈ {1,...T}. We typically consider k-mers of length 6 to 10 with a limited number of degenerate positions, assuming that a motif has a conserved core of at least three to five nucleotides, where binding tends to occur. A match of motif m_j _in sequence region s_i _is given by the binary indicator variable x_ij_. If we denote the number of motif occurrences in ${\left\{{\text{s}}_{\text{i}}\right\}}_{i=1}^{n}$ by:

then:

$$\begin{array}{l} {\text{e}}_{\text{j}}=\frac{1}{{\text{n}}_{\text{j}}}\sum_{\text{i}:{\text{x}}_{\text{ij}}=1}{\text{y}}_{\text{i}}^{*},\;{\hat{\sigma }}_{\text{j}}^{2}=\frac{{\hat{\sigma }}^{2}}{{\text{n}}_{\text{j}}}\\ {\text{S}}_{\text{j}}^{\text{cERMIT}}={\text{A}}_{\text{j}}\times \frac{{\text{e}}_{\text{j}}}{{\hat{\sigma }}_{\text{j}}}\\ {\text{m}}_{\text{cERMIT}}^{*}=\underset{\text{j}\in \{1,..,\text{T}\}}{\arg\;\max}\;S_{\text{j}}^{\text{cERMIT}} \end{array}$$

where ${\text{m}}_{\text{cERMIT}}^{\text{*}}$ denotes the top predicted motif using the strategy described in Georgiev *et al*. [8].

Upon re-analysis of a previous benchmark yeast ChIP dataset [8], we noticed improved prediction accuracy (an additional approximately 17% of successfully recovered motifs) by using the full set of 7-mer non-degenerate oligomers (instead of 5-mers) as a starting point for the cERMIT motif search (improved motif space exploration), and requiring a minimum target set size of 5% (improved motif score stability), and therefore adopted these search parameters for all sequence-specific PAR-CLIP analyses in this study. In addition to an RNA binding profile description in the form of a position-specific scoring matrix, we report the set of predicted motif occurrences in decreasing order of binding evidence for the corresponding sequence region, in order to facilitate downstream analyses of biological function and potential regulatory network reconstruction. For visualization, resulting motifs were represented as logos using the WebLogo tool [46].

### microRNA enrichment analysis

With some notable exceptions, post-transcriptional regulation of miRNAs is largely mediated by sequence complementarity of the canonical miRNA 5' seeds to mRNA transcripts [20,21]. Argonaute pull-down data generated by PAR-CLIP protocol provides the ensemble of such targeted transcripts in the cell. To identify highly abundant mRNA transcripts, complementary to canonical seeds of known/highly expressed miRNAs, we implemented a tailored version of cERMIT, mEAT. In mEAT, we limit the search for enriched functional sequence motifs to a pre-specified list of known miRNAs-for example, as defined in miRBase-and evaluate over all those miRNAs instead of a greedy *de novo *motif search. When additional information on miRNA expression is known, this list can be further restricted to the subset of (top) expressed miRNAs. In the spirit of previously published work [47-49], we used a linear regression model for the interaction site binding evidence, which closely resembles the cERMIT scoring strategy described above [8].

Let the regression coefficient for motif m_j _be denoted as *β*_j_, then a simple linear regression model for the binding evidence is:

$${\text{ y}}_{{}_{\text{i}}}^{\text{*}}\;={\text{x}}_{\text{ij}}\beta_{\text{j}}+\varepsilon_{\text{i}}\text{, }\varepsilon_{\text{i}}\sim \text{N(0, }\sigma^{\text{2}}\text{)}$$

Using the classical ordinary least squares (OLS) estimator for the regression coefficient:

$${\sim \hat{\beta }}_{\text{j}}^{\text{OLS}}\;=\frac{\text{1 }}{{\text{n}}_{\text{j}}}\overset{\text{n}}{\underset{\text{i}=\text{1}}{\sum }}1\left[{\text{x}}_{\text{ij}}=\text{1}\right]\times {\text{y}}_{\text{i}}^{\text{*}}$$

we define the motif enrichment score to be:

$${\text{S}}_{\text{j}}^{\text{reg}}=\;\frac{{\hat{\beta }}_{\text{j}}^{\text{OLS}}}{{\hat{\sigma }}_{\text{j}}}=\frac{\text{1}}{{\text{A}}_{\text{j}}}\times {\text{S}}_{\text{j}}^{\text{cERMIT}}$$

which results in the top prediction:

$${\text{ m}}_{\text{reg}}^{\text{*}}\;=\;\underset{\text{j}\in \text{\{1,}..\text{,T\}}}{\text{arg max }}{\text{ S}}_{\text{j}}^{\text{reg}}$$

In the typical scenario, in which the size of the motif target set is small relative to the number of all sequence regions (n_j _< < n), this results in A_j _≈ 1, and ${\text{S}}_{\text{j}}^{\text{reg}}\approx {\text{S}}_{\text{j}}^{\text{cERMIT}}$. We extended this basic model to a regression approach in which the evidence for each interaction site is modeled as a linear combination of a binary indicator variable for the presence of a motif, and additional confounder covariates with some added noise. We here use a single type of confounder, the di-nucleotide counts in each sequence region, and represent the miRNA by the list of seven canonical seed types mentioned above. Using this confounder is motivated by using observed PAR-CLIP interaction sites as inputs, and allows us to control for the locally higher AU content around miRNA target sites in 3' UTRs compared to the overall transcript background.

More generally, consider p confounders and define the matrix of covariates to be Z_j _= (x_j_, c_1_,...c_p_) where x_j _denotes the column vector of binary indicators of the j-th miRNA seed type, j ∈ {1,..., 7}, and c_k _∈ ℜ^n^, k ∈ {1,..., p} denote the di-nucleotide counts (hence p = 16), mean-centered and normalized to have sample standard deviation of 1. With corresponding regression coefficients β_j _= (β_1j_,..., β_p+ij_)^T ^∈ ℜ^p+1 ^the linear regression model for the binding evidence becomes (using matrix notation):

$${\text{Y}}^{*}={\text{Z}}_{\text{j}}\beta_{\text{j}}+\varepsilon ,\;\varepsilon \sim \text{N}\left(0,\;\sigma {\text{I}}_{\text{n}\times \text{n}}\right)$$

We estimate the regression coefficients using OLS, ${\hat{\beta }}_{\text{j}}\;={\left({\text{Z}}_{\text{j}}^{\text{T}}{\text{Z}}_{\text{j}}\right)}^{-\text{1}}{\text{Z}}_{\text{j}}^{\text{T}}{\text{Y}}^{\text{*}}\text{, }{\hat{\Sigma }}_{{\hat{\beta }}_{\text{j}}}={\sim \hat{\sigma }}^{\text{2}}{\left({\text{Z}}_{\text{j}}^{\text{T}}{\text{Z}}_{\text{j}}\right)}^{-\text{1}}$, which can be expected to produce stable results in the typical setting with a large number of clusters. An independent regression model is fit for each miRNA seed type for j ∈ {1,..., 7}, and the miRNA score is defined as the average score of all (positively scoring) seed types:

$${\text{S}}_{\text{j}}^{\text{REG}}=\frac{{\sim \hat{\beta }}_{\text{1j}}}{\sqrt{{\text{(}{\sim \hat{\Sigma }}_{\sim \hat{\beta }j}\text{)}}_{\text{11}}}}\;\Rightarrow {\text{S}}^{\text{REG}}\;=\;\frac{\text{1}}{\sum_{\text{j}}{\text{1[S}}_{\text{j}}^{\text{REG}}>\text{0]}}\overset{\text{7}}{\underset{\text{j}=\text{1}}{\sum }}{\text{1[S}}_{\text{j}}^{\text{REG}}>\text{0]}{\text{ S}}_{\text{j}}^{\text{REG}}\;$$.

Alternative definitions (for example, maximum score, sum of scores) and scoring schemes (principal components regression) produced similar results, yet required additional assumptions (for example, specification of the number of components, and so on). An additional filtering step helps avoiding inflated miRNA scores due to random chance. A set of randomized scores is generated by permuting the binding evidence B (default of 100) times, with scores S^REG(b)^, b ∈ {1,..., B} estimated using OLS. From these scores, we fit an empirical null distribution using a Gaussian parametric model; the observed miRNA score S^REG ^is considered significant if it is found to be larger than a user-specified number of standard deviation relative to the mean of the null distribution (default of 3 standard deviations). The corresponding *P*-value can be used as a guide to the significance of the reported individual miRNA enrichment scores.

Many of the top scoring miRNAs will have canonical seeds that are very similar (for example, varying in a single flanking position). As a result, their matches to mRNA target sequences and resulting enrichment scores are too similar to be distinctive. For this reason, we add a post-processing step that clusters miRNAs with highly similar seeds around 'cluster centers' defined to be distinct miRNAs with the highest score that are not part of an existing cluster. We initialize the clustering procedure by setting the first 'cluster center' to be the top scoring miRNA in the whole set of candidates. When deciding upon cluster membership, two miRNAs are considered to be similar to each other if they share a canonical motif that is at least seven consecutive nucleotides long.

## Abbreviations

4SU, 4-thiouridine; AGO, Argonaute; CCR, crosslink-centered region; cERMIT, conserved Evidence-Ranked Motif Identification Tool; CLIP, crosslinking and immunoprecipitation; IGF2BP1, Insulin-like growth factor 2 binding protein 1; mEAT, miRNA enrichment analysis tool; miRNA, microRNA; OLS, ordinary least squares; PAR-CLIP, photoactivatable-ribonucleoside-enhanced crosslinking and immunoprecipitation; PUM2, Pumilio2; QKI, Quaking; RBP, RNA binding protein; RISC, RNA-induced silencing complex; UTR, untranslated region.

## Supplementary Material

Additional file 1 **Correlation of read numbers and number of T = > C conversion events observed in PARalyzer interaction sites**. The number of observed T = > C conversions strongly correlates with the total number of reads. Data are taken from the Argonaute 1 to 4 dataset. Click here for file

Additional file 2 **Number of sites per nucleotide in PARalyzer interaction sites that fall within intergenic regions compared to genic regions**. Click here for file

Additional file 3 **Location of PARalyzer interaction sites under a more lenient mapping strategy**. Reads were mapped to the genome allowing up to three mismatches. The mismatches were not required to be a T = > C mismatch. **(a) **Genomic location of interaction sites that contain at least a single T = > C conversion event. **(b) **Genomic locations of interaction sites that contain T = > C conversions at a minimum of two separate locations. Click here for file

Additional file 4 **Signal-to-noise comparison between PARalyzer interaction sites and crosslink-centered regions**. **(a) **The log2 signal-to-noise for the top 20 expressed miRNAs in the Argonaute dataset for both PARalyzer-generated interaction sites and the Hafner *et al*. [7] CCRs found within coding regions. **(b) **Average log2 signal-to-noise ratio of window size 21 across all 361 miRNAs reported expressed in Hafner *et al*. [7], in the order of their expression rank. Click here for file

Additional file 5 **Sequence context at regulatory motifs for Argonaute (AGO) 1 to 4**. Non-redundant seed-matches in coding regions for the top 20 expressed miRNAs in the Argonaute dataset. 8 mer-m1 is a seed-match between the mRNA and nucleotides 1 to 8 of the miRNA seed sequence, 8 mer-A1 matches nucleotides 2 to 8 of the seed sequence paired with an A at position 1. 7 mer-1 m and 7 mer-A1 are similarly defined for nucleotides 1 to 7; 7 mer-m8 is a match utilizing nucleotides 2 to 8 of the seed sequence. 6 mer2-7 is a match utilizing nucleotides 2 to 7 of the seed sequence, and 6 mer3-8 utilizes nucleotides 3 to 8 of the sequence. Heatmap: nucleotide composition, relative to a uniform background, of each individual binding site found in the coding region of a gene. Barplot: likelihood of a T = > C conversion given that there is a 'T' at the given position. Unlike the heatmap, the barplot is not normalized by the number of reads mapping to an individual binding site. The horizontal dotted red line indicates the background conversion probability for all 'T's within the respective coding region. Click here for file

Additional file 6 **Quaking (QKI) motif prediction**. cERMIT calculated motif logo for QKI based on the PARalyzer generated interaction sites. For this analysis, we used interaction sites that contained at least five reads, mapped to a genic region, contained at least two T = > C conversions and did not overlap a repeat region. Click here for file

Additional file 7 **Pumilio2 (PUM2) motif prediction**. cERMIT calculated motif logo for PUM2 based on PARalyzer generated interaction sites. For this analysis, we used interaction sites that contained at least five reads, mapped to a genic region, contained at least two T = > C conversions and did not overlap a repeat region. Click here for file

Additional file 8 **Insulin-like growth factor 2 binding protein (IGF2BP1) motif prediction**. cERMIT calculated motif logo for IGF2BP1 based on PARalyzer generated interaction sites. For this analysis, we used interaction sites that contained at least five reads, mapped to a genic region, contained at least two T = > C conversions and did not overlap a repeat region. Click here for file

Additional file 9 **Effect of bandwidth parameter on signal-to-noise**. The signal-to-noise ratio is plotted for different bandwidth parameters as calculated from both the Pumilio2 and Quaking datasets. Interaction sites were required to fall within a genic region, contain two or more conversion events, and not overlap a repeat region. Click here for file

Additional file 10 **Summary of motif matches in the different PAR-CLIP datasets when using a minimum read depth of one read**. Click here for file
