# Supplementary figures and images for: PARalyzer: definition of RNA binding sites from PAR-CLIP short-read sequence data
Source: Genome Biol. 2011 Aug 18;12(8):R79. doi: 10.1186/gb-2011-12-8-r79 (PMC3302668; doi:10.1186/gb-2011-12-8-r79)

Read Count vs. Conversion Events by Interaction Site

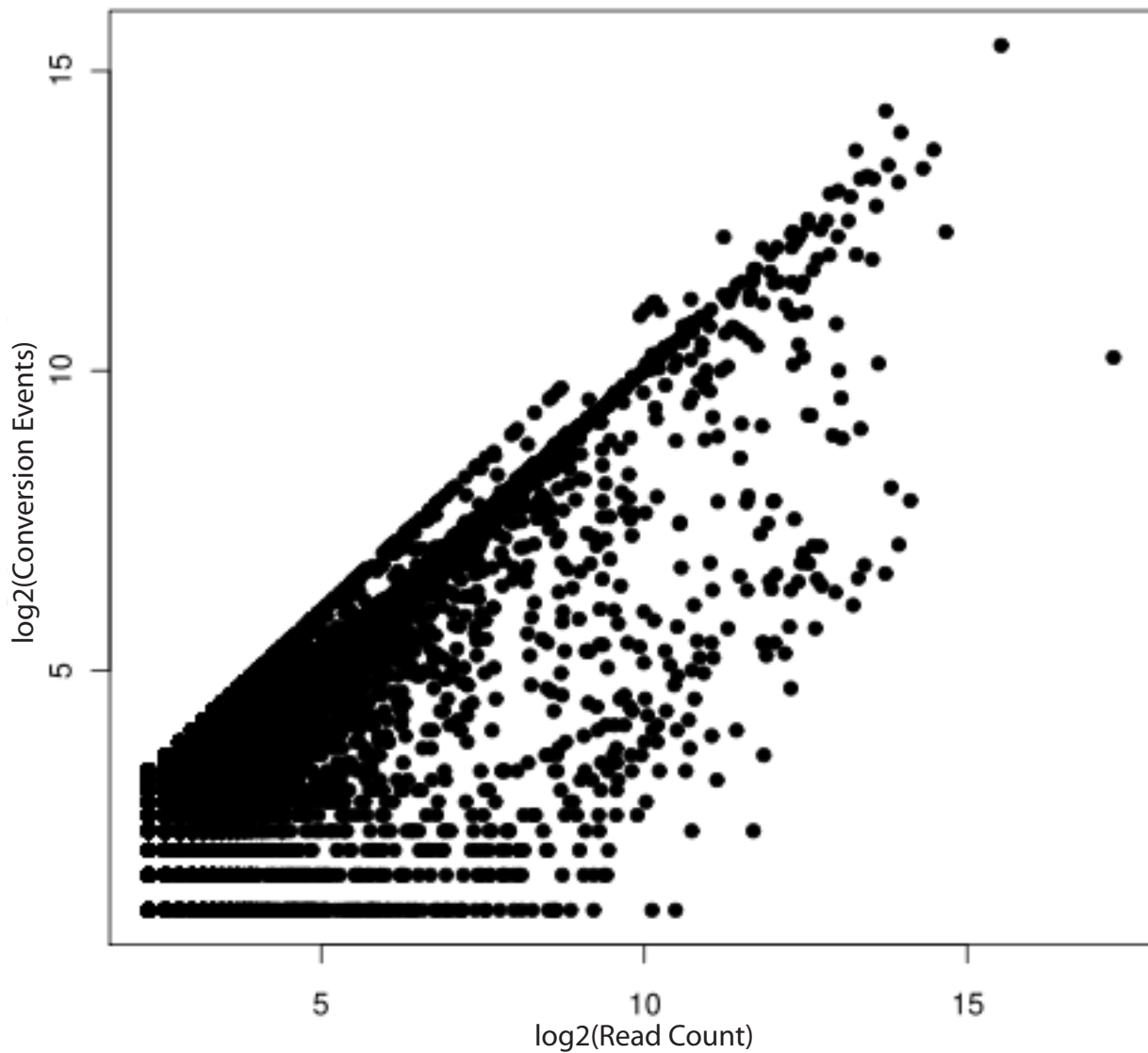

Supplement: Additional file 1 — Correlation of read numbers and number of T = > C conversion events observed in PARalyzer interaction sites. The number of observed T = > C conversions strongly correlates with the total number of reads. Data are taken from the Argonaute 1 to 4 dataset. [file gb-2011-12-8-r79-S1.PDF]

**A**

# Argonaute1-4

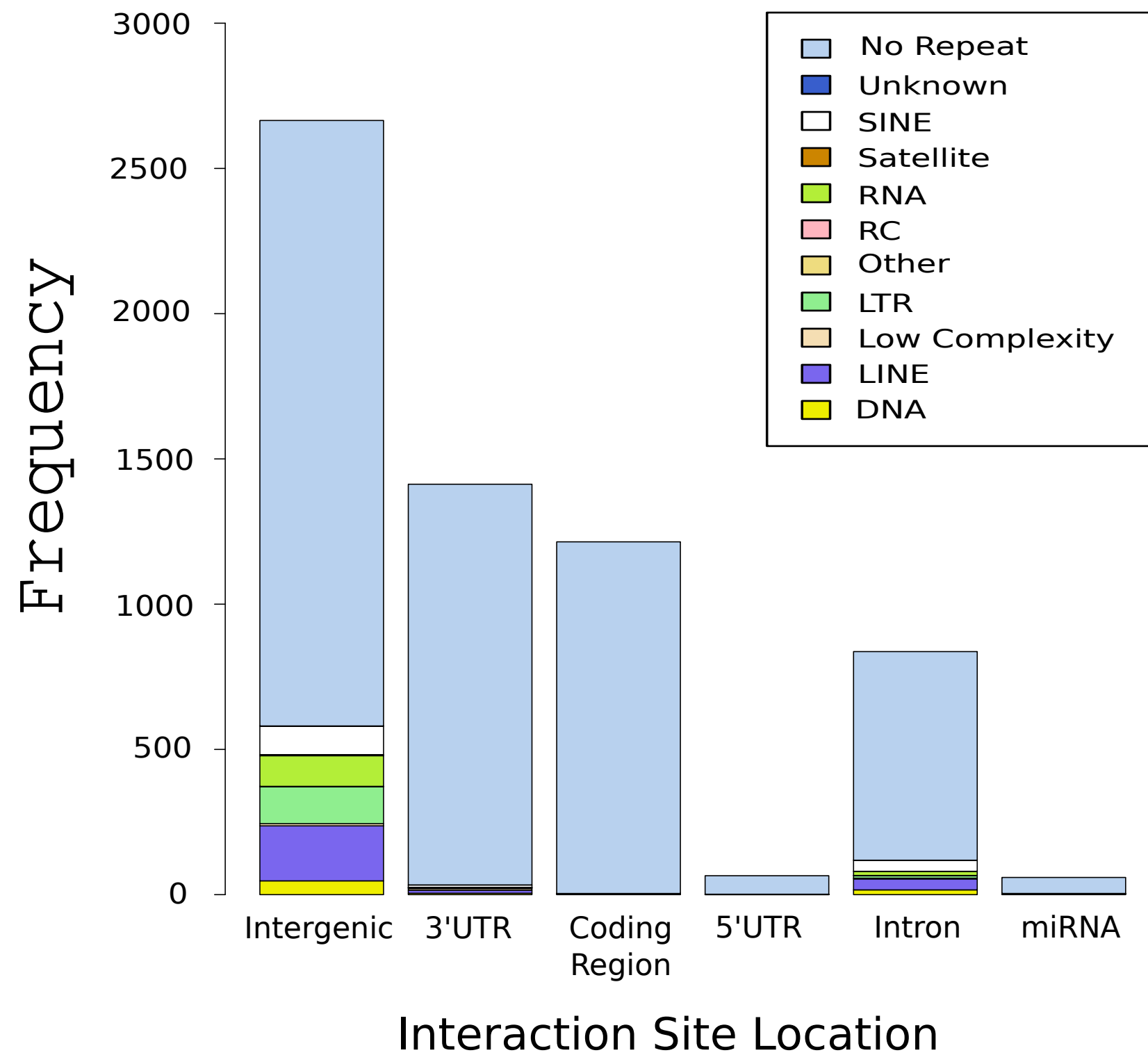

# B

# Argonaute1-4

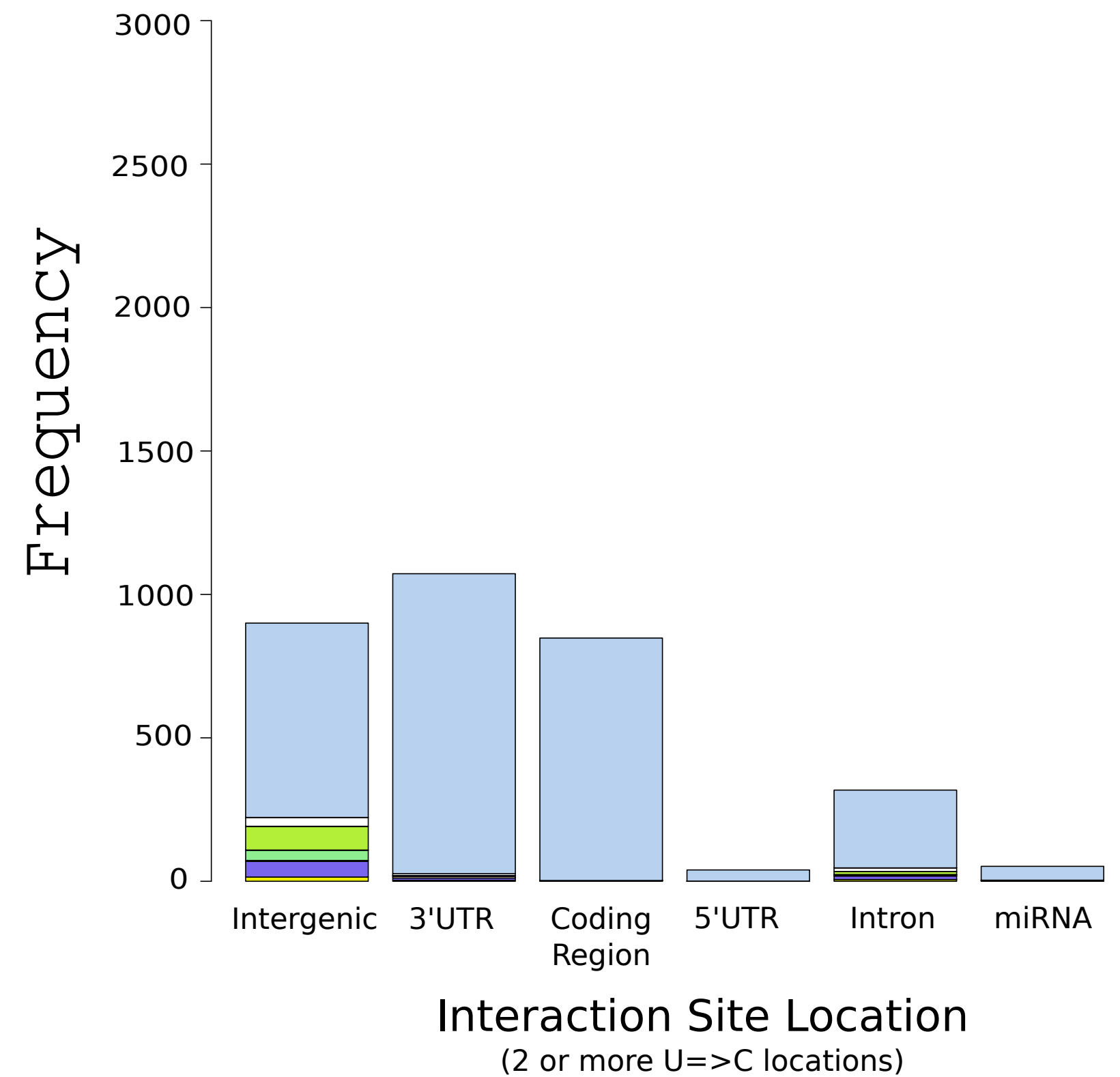

Supplement: Additional file 3 — Location of PARalyzer interaction sites under a more lenient mapping strategy. Reads were mapped to the genome allowing up to three mismatches. The mismatches were not required to be a T = > C mismatch. (a) Genomic location of interaction sites that contain at least a single T = > C conversion event. (b) Genomic locations of interaction sites that contain T = > C conversions at a minimum of two separate locations. [file gb-2011-12-8-r79-S3.PDF]

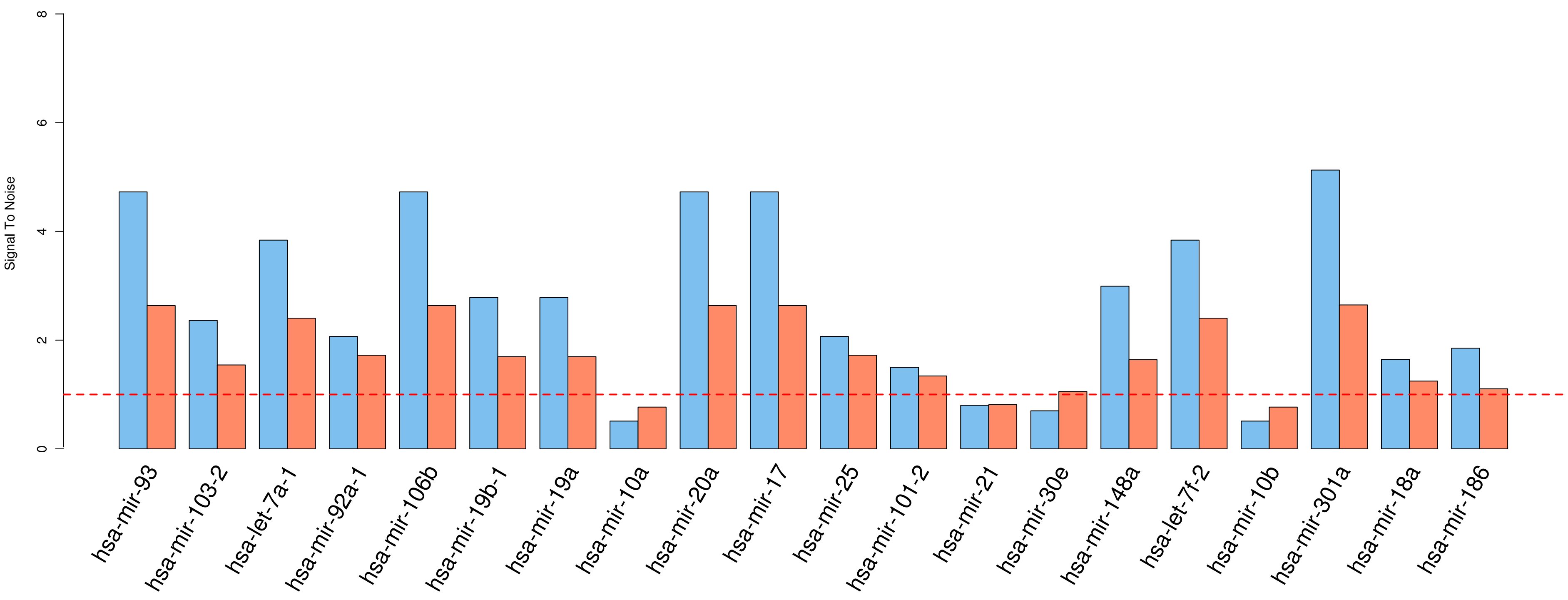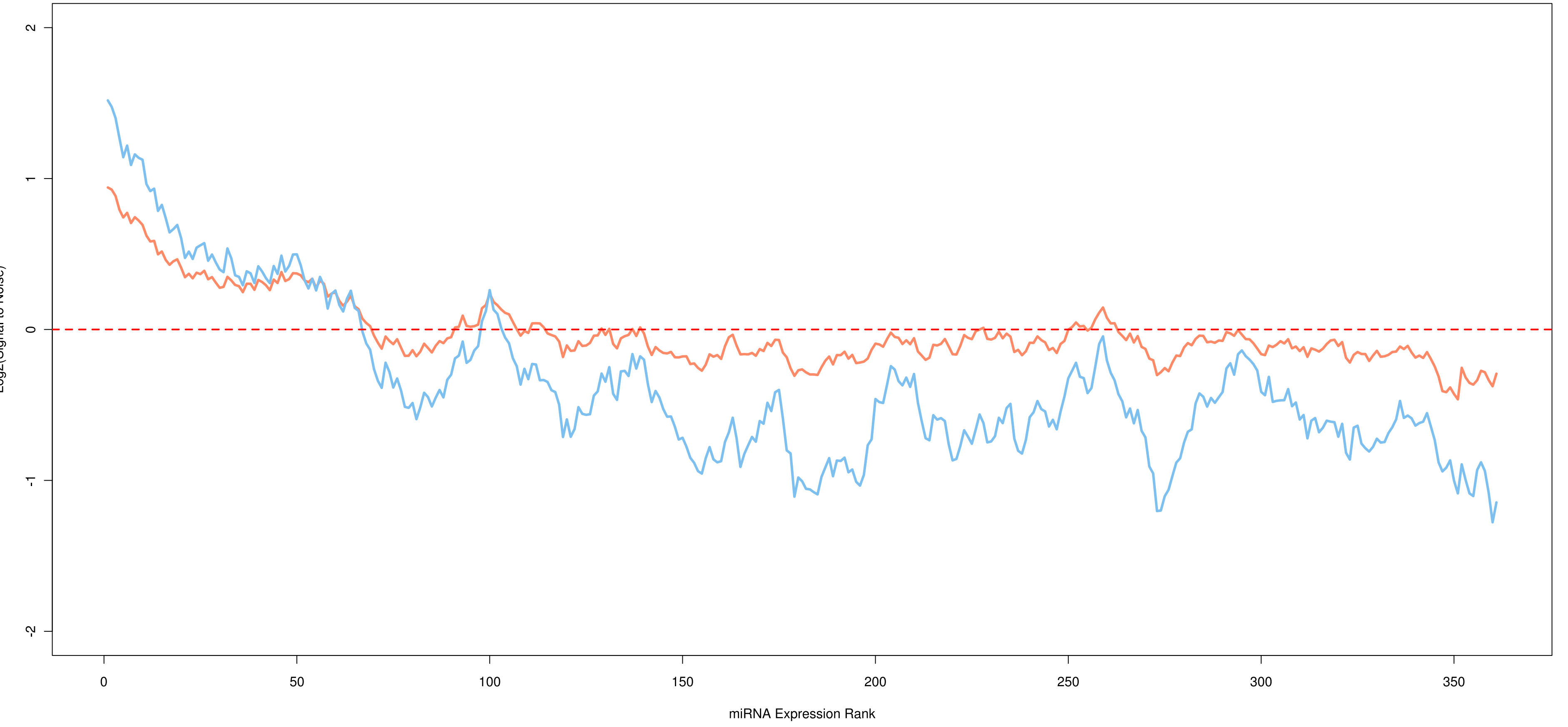

Supplement: Additional file 4 — Signal-to-noise comparison between PARalyzer interaction sites and crosslink-centered regions. (a) The log2 signal-to-noise for the top 20 expressed miRNAs in the Argonaute dataset for both PARalyzer-generated interaction sites and the Hafner et al. [7] CCRs found within coding regions. (b) Average log2 signal-to-noise ratio of window size 21 across all 361 miRNAs reported expressed in Hafner et al. [7], in the order of their expression rank. [file gb-2011-12-8-r79-S4.PDF]

# Argonaute1-4

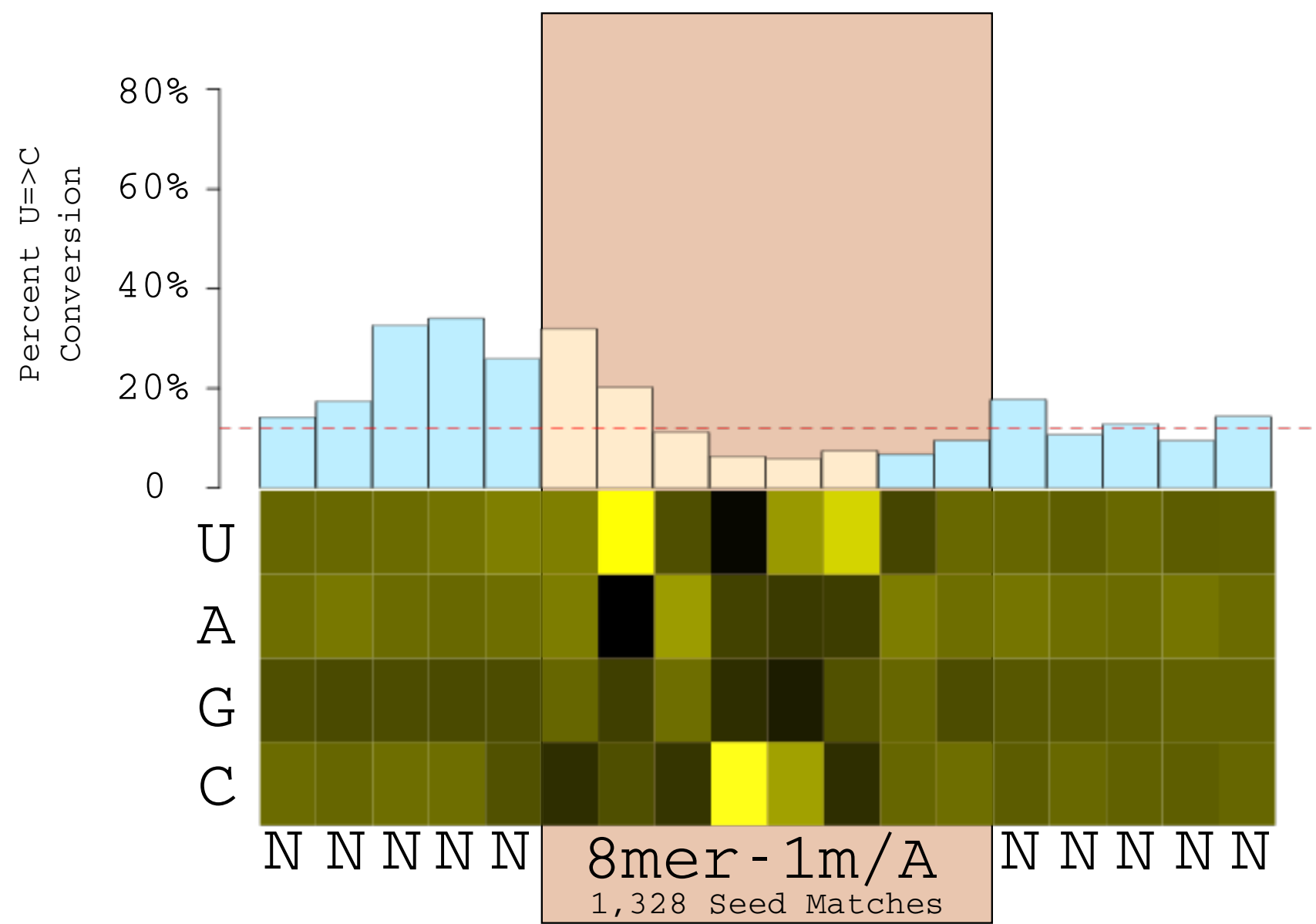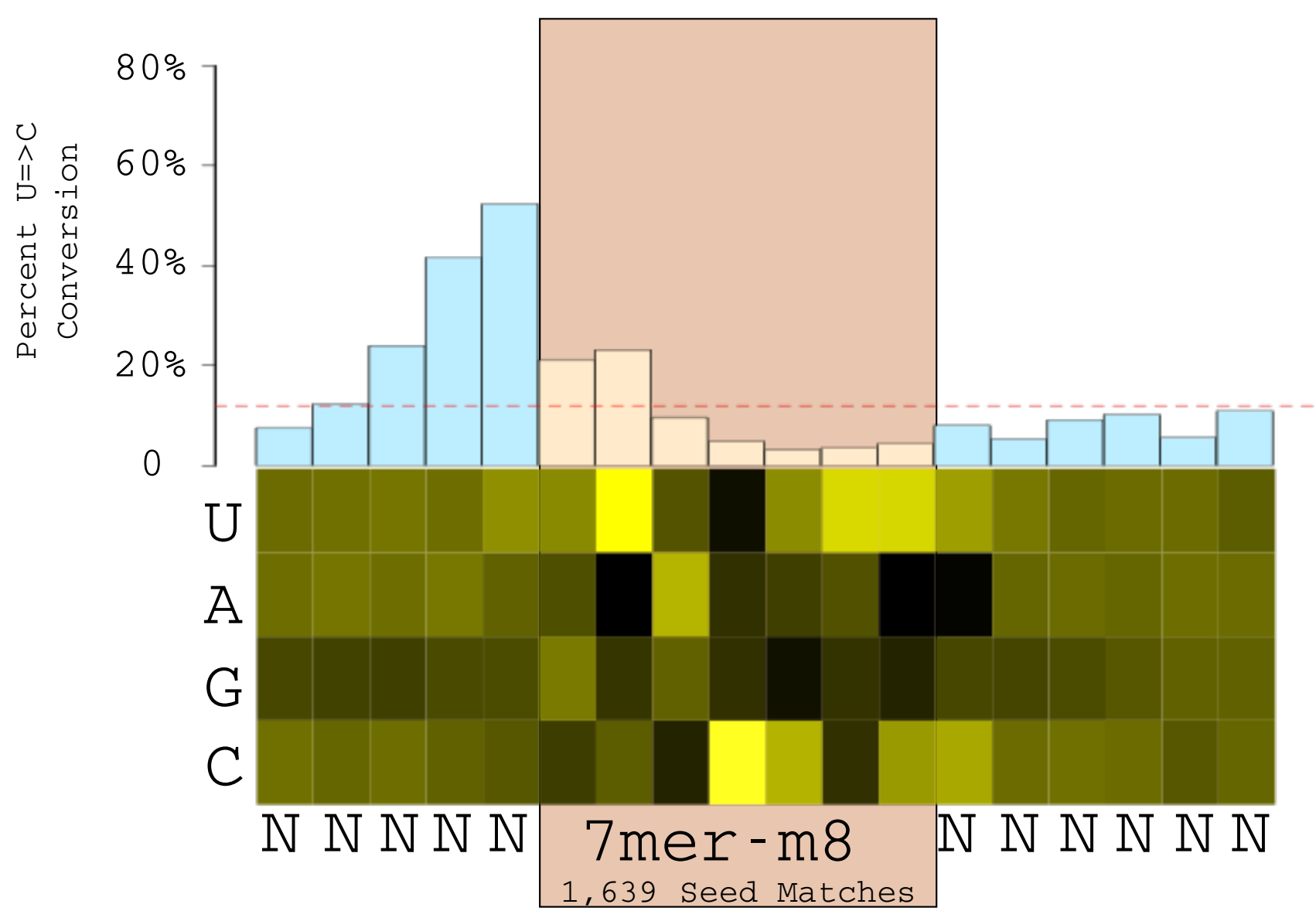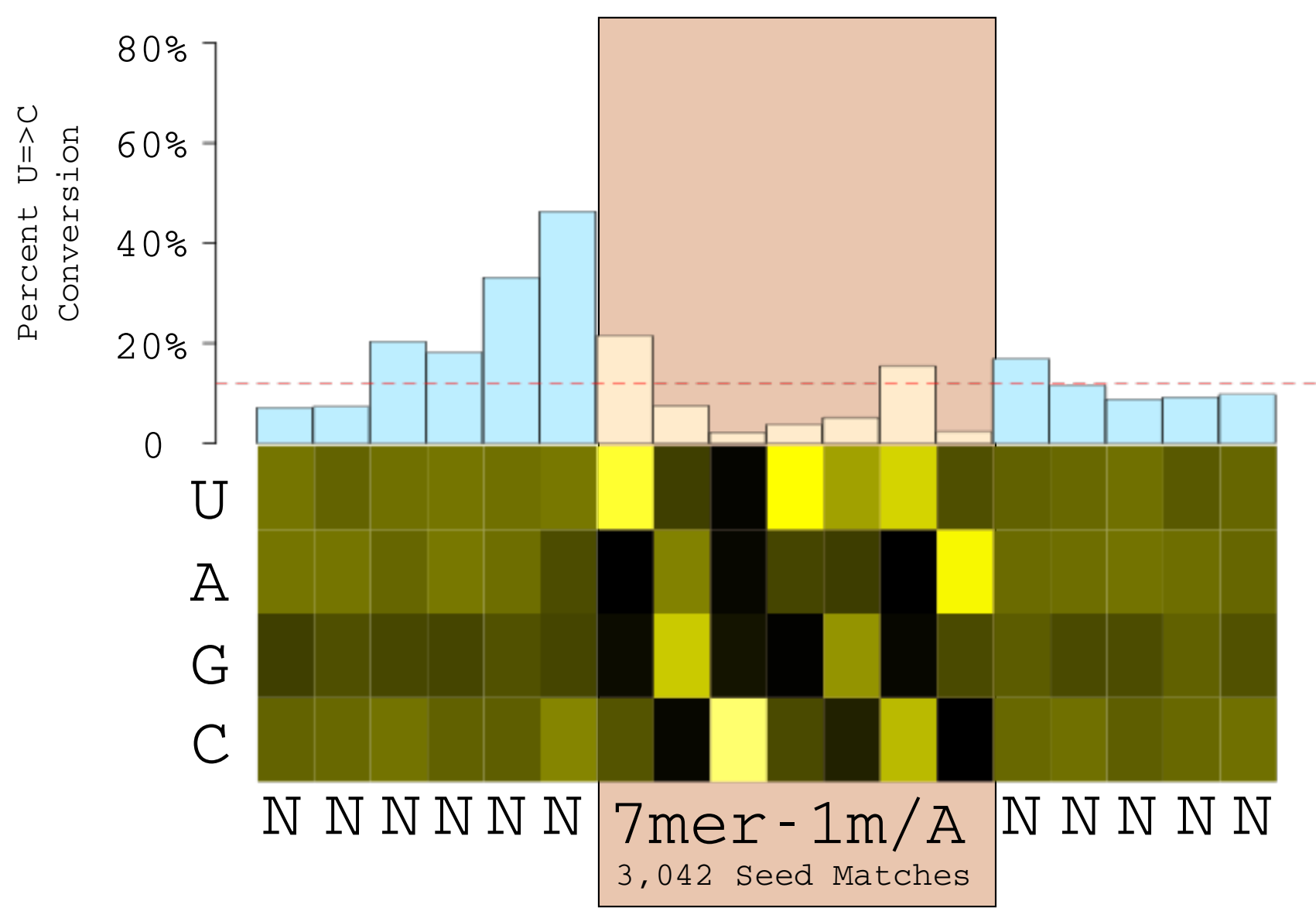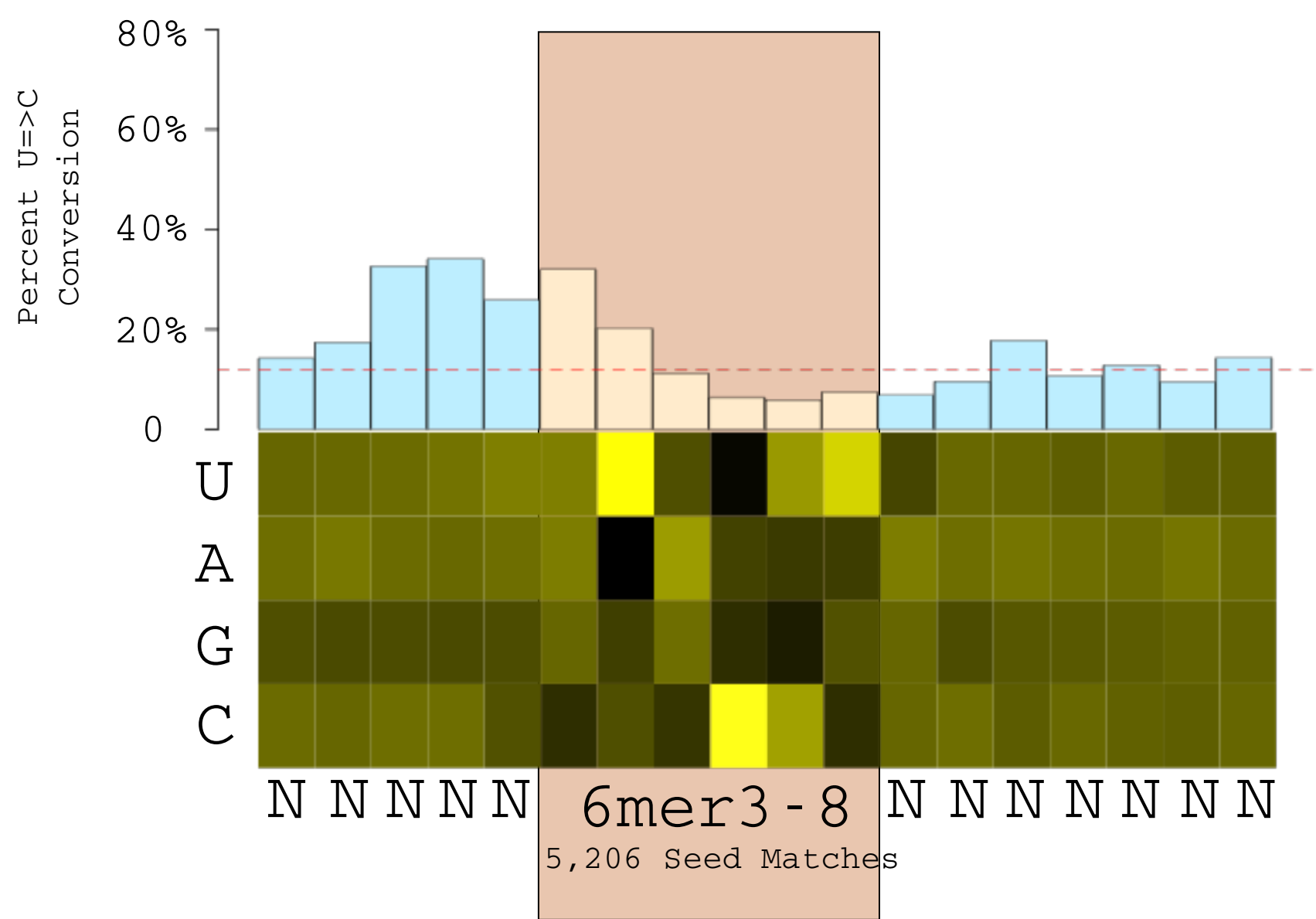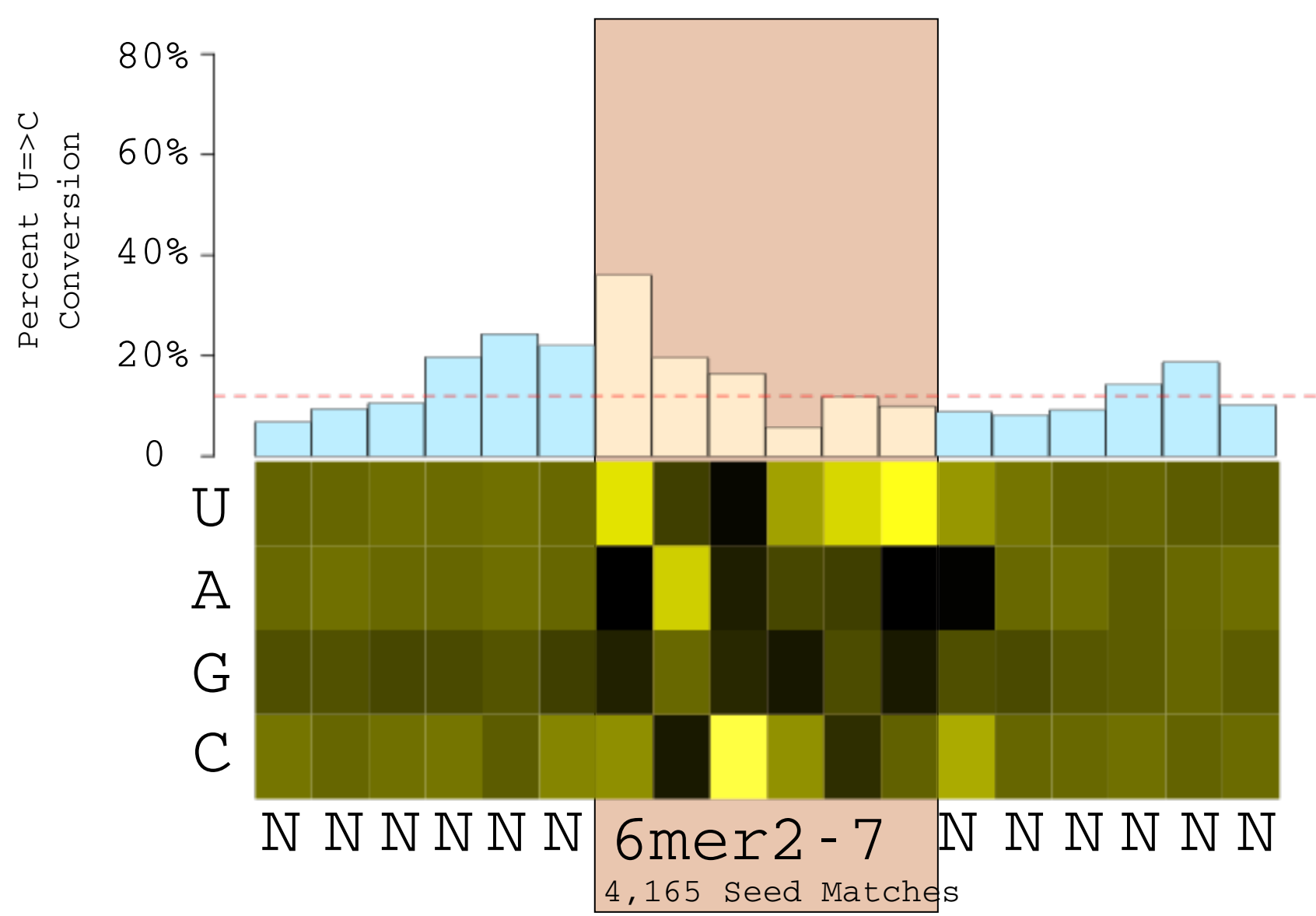

Supplement: Additional file 5 — Sequence context at regulatory motifs for Argonaute (AGO) 1 to 4. Non-redundant seed-matches in coding regions for the top 20 expressed miRNAs in the Argonaute dataset. 8 mer-m1 is a seed-match between the mRNA and nucleotides 1 to 8 of the miRNA seed sequence, 8 mer-A1 matches nucleotides 2 to 8 of the seed sequence paired with an A at position 1. 7 mer-1 m and 7 mer-A1 are similarly defined for nucleotides 1 to 7; 7 mer-m8 is a match utilizing nucleotides 2 to 8 of the seed sequence. 6 mer2-7 is a match utilizing nucleotides 2 to 7 of the seed sequence, and 6 mer3-8 utilizes nucleotides 3 to 8 of the sequence. Heatmap: nucleotide composition, relative to a uniform background, of each individual binding site found in the coding region of a gene. Barplot: likelihood of a T = > C conversion given that there is a 'T' at the given position. Unlike the heatmap, the barplot is not normalized by the number of reads mapping to an individual binding site. The horizontal dotted red line indicates the background conversion probability for all 'T's within the respective coding region. [file gb-2011-12-8-r79-S5.PDF]

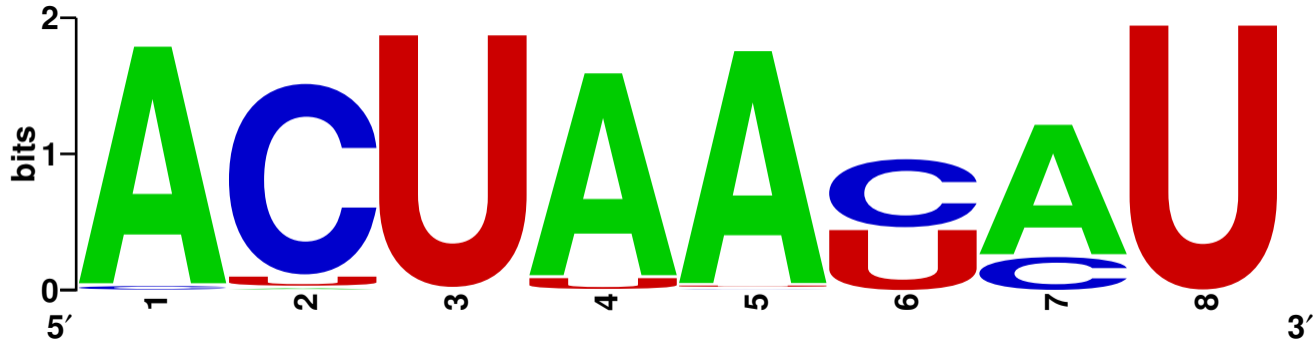

Supplement: Additional file 6 — Quaking (QKI) motif prediction. cERMIT calculated motif logo for QKI based on the PARalyzer generated interaction sites. For this analysis, we used interaction sites that contained at least five reads, mapped to a genic region, contained at least two T = > C conversions and did not overlap a repeat region. [file gb-2011-12-8-r79-S6.PDF]

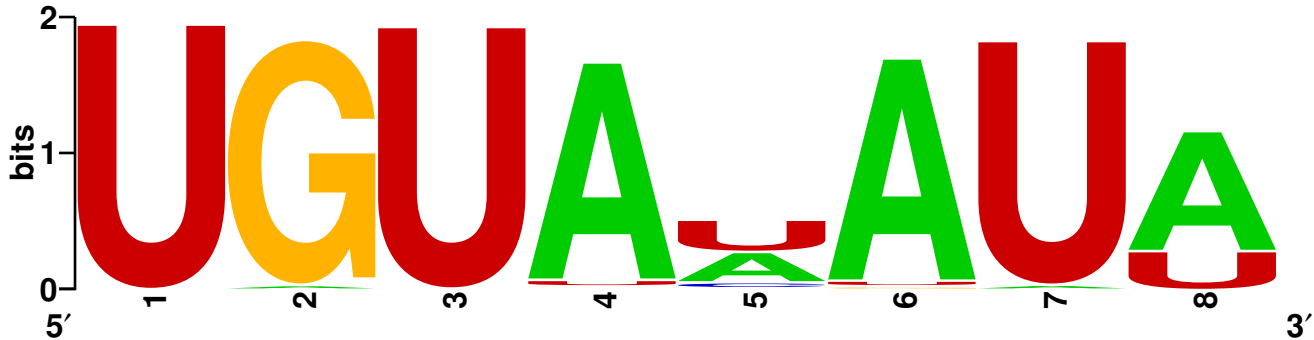

Supplement: Additional file 7 — Pumilio2 (PUM2) motif prediction. cERMIT calculated motif logo for PUM2 based on PARalyzer generated interaction sites. For this analysis, we used interaction sites that contained at least five reads, mapped to a genic region, contained at least two T = > C conversions and did not overlap a repeat region. [file gb-2011-12-8-r79-S7.PDF]

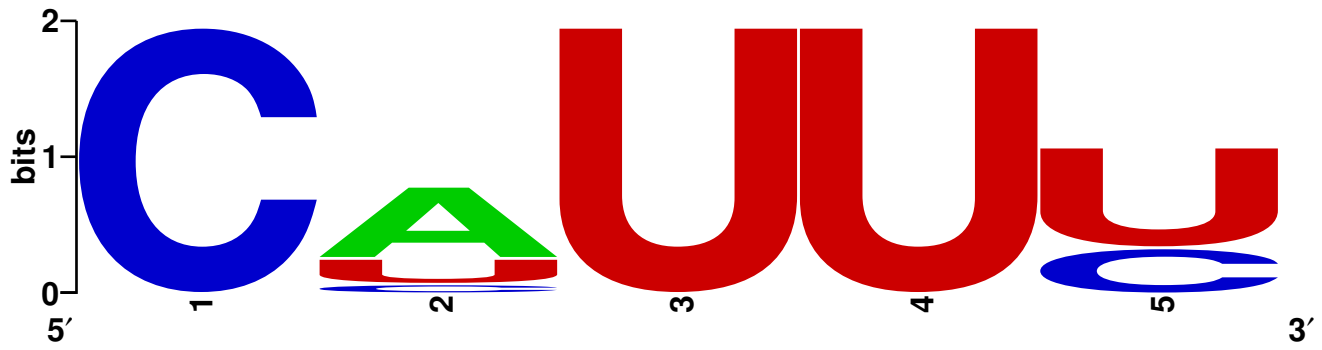

Supplement: Additional file 8 — Insulin-like growth factor 2 binding protein (IGF2BP1) motif prediction. cERMIT calculated motif logo for IGF2BP1 based on PARalyzer generated interaction sites. For this analysis, we used interaction sites that contained at least five reads, mapped to a genic region, contained at least two T = > C conversions and did not overlap a repeat region. [file gb-2011-12-8-r79-S8.PDF]

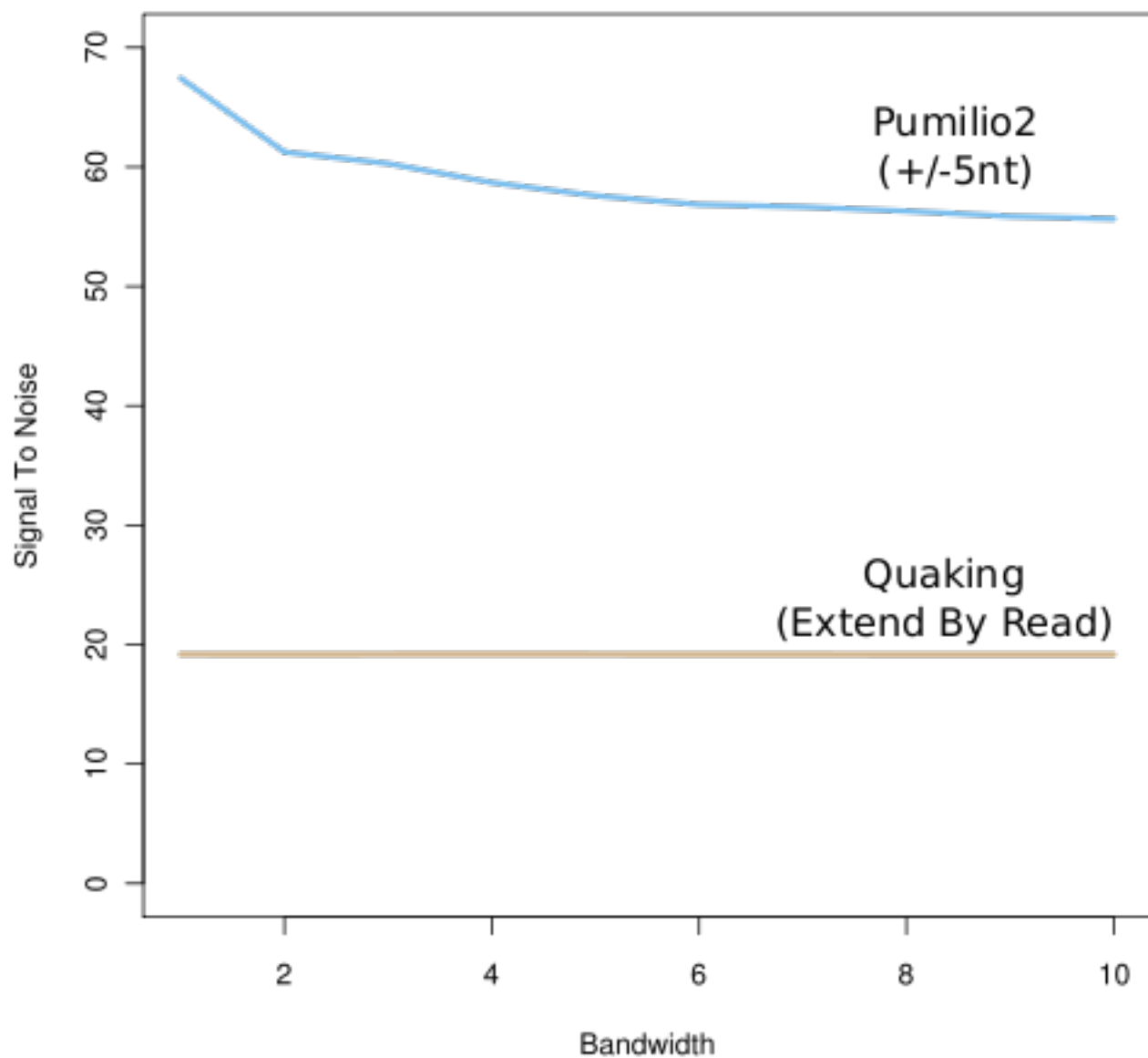

Supplement: Additional file 9 — Effect of bandwidth parameter on signal-to-noise. The signal-to-noise ratio is plotted for different bandwidth parameters as calculated from both the Pumilio2 and Quaking datasets. Interaction sites were required to fall within a genic region, contain two or more conversion events, and not overlap a repeat region. [file gb-2011-12-8-r79-S9.PDF]
