# Supplementary material for: PARalyzer: definition of RNA binding sites from PAR-CLIP short-read sequence data
Source: Genome Biol. 2011 Aug 18;12(8):R79. doi: 10.1186/gb-2011-12-8-r79 (PMC3302668; doi:10.1186/gb-2011-12-8-r79)
Supplement: Additional file 2 — Number of sites per nucleotide in PARalyzer interaction sites that fall within intergenic regions compared to genic regions. [file gb-2011-12-8-r79-S2.DOC]

**Additional File 2**: Number of sites per nucleotide in PARalyzer interaction sites that fall within intergenic regions as compared to genic regions.

|  | Number of Motif Matches | Total Nucleotides |
| --- | --- | --- |
| AGO |  |  |
| PARalyzer (genic) | 3,933 | 207,334 |
| Hafner *et al.* (genic) | 4,106 | 301,227 |
| PARalyzer (Intergenic) | 1,062 | 79,645 |
| PUM2 |  |  |
| PARalyzer (genic) | 1,262 | 127,168 |
| Hafner *et al.* (genic) | 1,371 | 200,228 |
| PARalyzer (Intergenic) | 336 | 4,258 |
| QKI |  |  |
| PARalyzer (genic) | 3,001 | 155,237 |
| Hafner *et al.* (genic) | 2,593 | 127,201 |
| PARalyzer  (Intergenic) | 885 | 75,659 |
| IGF2BP1 |  |  |
| PARalyzer (genic) | 31,507 | 1,718,152 |
| Hafner *et al.* (genic) | 51,429 | 3,739,750 |
| PARalyzer (Intergenic) | 5,921 | 265,957 |

Summary of the motif matches for PUM2, QKI, and IGF2BP1 were generated from the analysis of the full transcript of all genes, including 5'UTRs, 3'UTRs, introns and coding regions as compared to PARalyzer interaction sites that fall within intergenic regions. Interaction sites were required to contain at least 2 T=>C conversion events and not overlap a known repeat element.
