# Supplementary material for: PARalyzer: definition of RNA binding sites from PAR-CLIP short-read sequence data
Source: Genome Biol. 2011 Aug 18;12(8):R79. doi: 10.1186/gb-2011-12-8-r79 (PMC3302668; doi:10.1186/gb-2011-12-8-r79)
Supplement: Additional file 10 — Summary of motif matches in the different PAR-CLIP datasets when using a minimum read depth of one read. [file gb-2011-12-8-r79-S10.DOC]

**Additional File 10:** Summary of motif matches in the different PAR-CLIP datasets (minimum read depth of 1 read).

|  | Number of Motif Matches | Total Nucleotides in Clusters | Signal-to-Noise |
| --- | --- | --- | --- |
| Argonaute  (top 20 expressed miRNAs) |  |  |  |
| PARalyzer | 5,228 | 322,116 | 2.29 |
| Hafner *et al.* (CCRs) | 4,106 | 301,227 | 1.92 |
| Background (3'UTRs) | 131,741 | 18,602,068 | -- |
| PUM2 |  |  |  |
| PARalyzer | 1,363 | 142,267 | 58.20 |
| Hafner *et al.* | 1,371 | 200,228 | 41.59 |
| Background | 113,478 | 689,309,457 | -- |
| QKI |  |  |  |
| PARalyzer | 3,121 | 161,117 | 19.23 |
| Hafner *et al.* | 2,593 | 127,201 | 20.24 |
| Background | 694,229 | 689,309,457 | -- |
| IGF2BP1 |  |  |  |
| PARalyzer | 48,079 | 2,820,586 | 1.24 |
| Hafner *et al.* | 51,429 | 3,739,750 | 1.01 |
| Background | 9,343,410 | 689,309,457 | -- |

The Argonaute results are specific to only the 3'UTR region and contain only non-redundant seed-matches. Summary of the motif matches for PUM2, QKI, and IGF2BP1 were generated from the analysis of the full transcript of all genes, including 5'UTRs, 3'UTRs, introns and coding regions.
